# Negative density-dependent dispersal in tsetse (*Glossina* spp): An artefact of inappropriate analysis

**DOI:** 10.1371/journal.pntd.0009026

**Published:** 2021-03-25

**Authors:** John W. Hargrove, John Van Sickle, Glyn A. Vale, Eric R. Lucas

**Affiliations:** 1 SACEMA, University of Stellenbosch, Stellenbosch, South Africa; 2 Department of Fisheries and Wildlife, Oregon State University, Corvallis, Oregon, United States of America; 3 Natural Resources Institute, University of Greenwich, Chatham, United Kingdom; 4 Liverpool School of Tropical Medicine, Liverpool, United Kingdom; University of Texas Southwestern Medical School, UNITED STATES

## Abstract

Published analysis of genetic material from field-collected tsetse (*Glossina* spp, primarily from the Palpalis group) has been used to predict that the distance (*δ*) dispersed per generation increases as effective population densities (*D*_*e*_) decrease, displaying negative density-dependent dispersal (NDDD). Using the published data we show this result is an artefact arising primarily from errors in estimates of *S*, the area occupied by a subpopulation, and thereby in *D*_*e*_. The errors arise from the assumption that *S* can be estimated as the area ($\hat{S}$) regarded as being covered by traps. We use modelling to show that such errors result in anomalously high correlations between $\hat{\delta }$ and $\hat{S}$ and the appearance of NDDD, with a slope of -0.5 for the regressions of log($\hat{\delta }$) on log(${\hat{D}}_{e}$), even in simulations where we specifically assume density-independent dispersal (DID). A complementary mathematical analysis confirms our findings. Modelling of field results shows, similarly, that the false signal of NDDD can be produced by varying trap deployment patterns. Errors in the estimates of *δ* in the published analysis were magnified because variation in estimates of *S* were greater than for all other variables measured, and accounted for the greatest proportion of variation in $\hat{\delta }$. Errors in census population estimates result from an erroneous understanding of the relationship between trap placement and expected tsetse catch, exacerbated through failure to adjust for variations in trapping intensity, trap performance, and in capture probabilities between geographical situations and between tsetse species. Claims of support in the literature for NDDD are spurious. There is no suggested explanation for how NDDD might have evolved. We reject the NDDD hypothesis and caution that the idea should not be allowed to influence policy on tsetse and trypanosomiasis control.

## Author summary

Published analysis of genetic material from field-sampled tsetse (*Glossina* spp) has been used to suggest that, as tsetse population densities decrease, rates of dispersal increase–displaying negative density-dependent dispersal (NDDD), perhaps in all tsetse species. It is further suggested that tsetse control operations might, as a consequence of NDDD, unleash enhanced invasion of areas cleared of tsetse, prejudicing the long-term success of control campaigns. We demonstrate that NDDD in tsetse is an artefact consequent on multiple errors of analysis and interpretation. The most serious of these errors stems from a misunderstanding of the way in which traps sample tsetse, resulting in large errors in estimates of the areas covered by the traps, and occupied by the subpopulations being sampled. Our modelling studies show that these errors can produce the false signal of NDDD, even in situations where DID is assumed. Errors in census population estimates are made worse through failure to adjust for variations in trapping intensity, trap performance, and in capture probabilities between geographical situations, and between tsetse species. We reject the NDDD hypothesis and caution that the idea should not be allowed to influence policy on tsetse and trypanosomiasis control.

## Introduction

Using critical assumptions about gene flow, a model developed by Rousset [1], and analyses of trap samples of tsetse flies (*Glossina* spp), de Meeûs et al. [2] claimed to have found strong support for the hypothesis that the dispersal distance per generation, in tsetse, increases as a power function of decreasing population density. The claim was based on genetic analyses of material from five different species of tsetse, sampled using traps in ten studies in six different countries in West and East Africa [3–11]. De Meeûs et al. concluded that negative density-dependent dispersal (NDDD) probably applied to all tsetse species [2]. They predicted that mean dispersal distance (*δ*) would increase by 200-fold when the effective population density (*D*_*e*_) of adult tsetse decreased from about 24,000 to 1 per square kilometre, the order of density decline commonly associated with tsetse control operations [12]. This led them to warn that such control measures could unleash enhanced invasion of areas cleared of tsetse, so prejudicing the long-term success of tsetse control campaigns. That, in turn, prompted them to suggest the necessity of using "area-wide and/or sequential treatments of neighboring sites" to counter the added invasion, and they implied that small control operations risk doing more harm than good.

The term "area-wide" applied to control campaigns is jargon for the eradication of whole infestations of tsetse up to natural barriers to reinvasion and is commonly associated with recommendations for the use of the costly and complex sterile insect technique [13]. It would be dismaying if area-wide control were indeed necessary, since small-scale operations run by local communities offer an economically viable way forward [14–16]. Moreover, if NDDD were a reality in tsetse, then it would imply risk even for the many large and seemingly successful operations which have already cleared tsetse from tens of thousands of square kilometres, but which have fallen short of tackling the whole fly-belt [17]. The implication is that such signal successes are in danger of becoming disasters due to long-term evolution of increased invasion rates [18]. All of these considerations are of added importance since the protocol developed for tsetse by de Meeûs et al. [2], henceforth called the “NDDD protocol”, is potentially applicable to any trappable creature, including many insect vectors of neglected tropical diseases. Hence, if the protocol is valid it should be extended to all of these vectors to assess whether NDDD must be considered when formulating policies for their control.

Recognising the potential importance of the NDDD hypothesis, de Meeûs et al. called for it to be field tested [18]. The clearest and most direct means of doing this would be via a set of mark, release and recapture experiments to assess both the density of tsetse and their rate of displacement in a variety of locations before and after control. Unfortunately, such experiments would not only be prohibitively costly and protracted, but would also be effectively impossible–given the problem of obtaining meaningful numbers of recaptures in places where tsetse density is low. Hence, the only workable option for field tests would seem to be yet more genetic analyses of the sort already made [2], effectively involving nothing better than getting the analytical procedure to check itself.

Given the problems of conducting pertinent experiments, the only available means we have of checking the NDDD hypothesis is to examine closely the medley of procedures, assumptions, arguments and citations on which it depends. We note immediately that, whereas it was predicted that NDDD applies to all tsetse species [2], the evidence for it is based largely on tsetse of the Palpalis group. There was only one representative of the Morsitans group, *G*. *pallidipes*, and no Fusca group species. There seems, thus, to be no *a priori* support for the claim that NDDD probably applied to all tsetse [2]. Moreover, modelling studies have already shown that, even if the NDDD hypothesis were correct, threats to tsetse control would be minimal [19]. It remains to show, however, whether there is any valid evidence that NDDD in tsetse exists at all, and hence whether it and its associated protocol should feature in policies of tsetse research and control, or be addressed in the study, control and management of other creatures.

Accordingly, we dissect here the evidence adduced by de Meeûs et al. [2] in favour of NDDD in tsetse. We show that the methods they used to estimate parameters are subject to large errors, and that such errors create the false signal of NDDD, even in simulated populations where NDDD has been specifically proscribed. We stress that we make no explicit or implicit criticism of the model of Rousset [1], nor of the genetic analyses in any of the studies that generated the data used by de Meeûs et al. [2]. We simply enumerate the errors, inconsistencies and false signals that arise from the way in which the Rousset model, and the genetic data, have been used and interpreted.

## Methods

### The de Meeûs et al. procedure and its variables [2]

In designing the methods, we recognised that the main support for the NDDD hypothesis is built around Eq (1):

$$\delta \approx \frac{1}{\sqrt{\pi bD_{e}}}=\frac{\sqrt{S}}{\sqrt{\pi bN_{e}}}$$ (1)

where *δ* is the predicted dispersal distance per generation of a tsetse population, and *N*_*e*_ is the effective population size, roughly defined as the number of adults in a population that will leave a genetic signature to the next generation. *N*_*e*_ was estimated using linkage disequilibrium methods [2,20]. *S* is the surface area occupied by the effective population, and *b* is the slope of the linear regression of the genetic distance among subpopulations on the log-transformed geographic distance among those subpopulations. *D*_*e*_ is the effective population density, equal to *N*_*e*_/*S*. [2]. We will show that the insertion of highly uncertain values into Eq 1 can generate spurious negative correlations between the estimated values of *D*_*e*_ and *δ*. Here, and in what follows, we represent estimated, as opposed to the true, values of the variables by $\hat{S},\;{\hat{N}}_{e}$, ${\hat{D}}_{e}$, $\hat{b}$ and $\hat{\delta }$. Note that de Meeûs et al. did not measure $\hat{\delta }$: they simply calculated its value from Eq (1), using estimates $\hat{b}$ and ${\hat{N}}_{e}$ obtained from genetic analyses, and estimates $\hat{S}$ of the area occupied by the population, calculated from the disposition of the traps that sampled the population [2].

### Analysis plan

We first looked at relationships between all of the variables in Eq (1). When we found several counter-intuitive correlations, we provided an heuristic explanation of how these could result from measurement error. Mathematical analysis, and a simulation study, were then used to confirm that errors in ${\hat{D}}_{e}$ lead to a false signal of NDDD. This led to an investigation of the importance of the variable *S* in contributing to the errors in ${\hat{D}}_{e}$ and a demonstration that there were indeed serious errors in the estimation of *S* in the de Meeûs et al. study [2]. Finally, we used data from two of the ten field studies used in the development of the NDDD protocol, carrying out simulations that tested the idea that variations in trap deployment patterns, or the way in which such patterns were interpreted, could lead to the false signal of NDDD. Details follow of the methods used in executing the above plan.

#### Analysis of relationships observed in the de Meeûs et al. study [2]

To elucidate the relative importance of the variables involved in Eq 1 we first took logarithms of both sides to get Eq 2:

$$log(\hat{\delta })\approx -0.5\;log(\pi )+0.5\;log(\hat{S})-0.5\;log(\hat{N}e)-0.5\;log(\hat{b})$$ (2)

Then, using data kindly supplied by Dr Thierry de Meeûs (Table A in S1 Text and Table A in S1 Table), we looked for correlations among the variables in Eq (2).

#### How measurement errors generate a false signal of NDDD with a slope of -0.5

The above analysis showed some counter-intuitive correlations, so we investigated the possibility that these could result from measurement error. We provide an heuristic explanation for the way in which such errors lead to a false signal of NDDD, and support this with a mathematical analysis, full details of which are provided in S2 Text.

#### The false signal of NDDD in a simulated population with assumed density-independent dispersal (DID)

We next carried out a simulation exercise confirming that errors in measures of *D*_*e*_ lead naturally to a false signal of NDDD in a situation where we specifically assume DID. This is illustrated in S2 Table, which provides full details of the simulation procedure, carried out in Excel, which can be executed by the reader. We simulate a group of populations where *δ* is constant, *D*_*e*_ varies within some arbitrary range, and *b* is calculated from *δ* and *D*_*e*_ according to Eq (1). We use these true values of *D*_*e*_ in this simulated population to generate “estimates” of ${\hat{D}}_{e}$ with large error. For this, ${\hat{D}}_{e}$ is calculated as true *D*_*e*_ multiplied by some random factor between 0.2 and 5; these errors can be made additive instead, without consequence to the conclusion. For simplicity, we assume that $\hat{b}$ is estimated without error ($\hat{b}$ = *b*). We then calculate $\hat{\delta }$ using Eq (1), replicating the method used in the NDDD protocol [2].

#### The importance of $\hat{S}$ in determining the value of $\hat{\delta }$

In the previous section we investigated how errors in ${\hat{D}}_{e}$ can lead to a false signal of NDDD. Since ${\hat{D}}_{e}$ = ${\hat{N}}_{e}/\hat{S}$ errors in $\hat{S}$ can be a major contributor in errors in ${\hat{D}}_{e}$, unless these are exactly cancelled out by other errors in ${\hat{N}}_{e}$. Accordingly, we investigated the importance of *S* in determining the *δ* value predicted by Eq (2), relative to contributions from other terms. Since the equation has the form of a multiple linear regression, we borrowed a tool from regression to estimate, for the data from the ten studies used by de Meeûs et al. [2], the relative importance of each of the predictor variables, log(${\hat{N}}_{e}$), log($\hat{S}$), and log($\hat{b}$), in determining the predicted value of log($\hat{\delta }$). We measure “relative importance” by the percentage of the total variance in log($\hat{\delta }$) that is explained by variation in each of the three predictor variables. Since these three variables are inter-correlated across the ten studies, we used hierarchical partitioning [21–23] to estimate their relative importance. In this approach, one of the variables, say log($\hat{S}$), is added to each of the four possible regression models that contain neither, either, or both of the other two predictors. In each case, the increase in multiple *R*^*2*^ due to the addition of log($\hat{S}$) is recorded, and the average of the four increases is the estimated proportion of total variance in log($\hat{\delta }$) that is explained by log($\hat{S}$). For example, regression of log($\hat{\delta }$) on log(${\hat{N}}_{e}$) alone yields *R*^*2*^ = 30%. The addition of log($\hat{S}$) to this regression model improves *R*^*2*^ to 85%, an increase of 55%. Increases in *R*^*2*^ are likewise calculated when log($\hat{S}$) is added to the three other possible regression models involving the other two predictors: the null model, the model containing log($\hat{b}$) alone, and the model containing both log($\hat{b}$) and log(${\hat{N}}_{e}$). The mean of the four *R*^*2*^ increases is 65%. This procedure is then repeated for log($\hat{b}$), and again for log(${\hat{N}}_{e}$), to obtain the mean increase in *R*^*2*^, when each is added to the four regression models involving the other two predictors. Because log(${\hat{N}}_{e}$), log($\hat{S}$), and log($\hat{b}$) were used to predict values of log($\hat{\delta }$) in the first place, via Eq (2), they jointly explain 100% of its total variance. We applied hierarchical partitioning to the data, using either the *hier*.*part* or *relaimpo* package of the R language.

#### The importance of errors in $\hat{S}$ in creating a false signal of NDDD

The preceding analyses led to the demonstration of the central importance of $\hat{S}$ in determining the estimated value of *δ*. We then show that the true area (*S*) covered by a biological subpopulation bears no known relation to the area ($\hat{S}$) estimated to be covered by a set of traps.

#### Simulations from field studies illustrating false signals of NDDD

Finally, we check the validity of the field estimates of population densities and resulting dispersal distances reported by de Meeûs et al. [2]. For each of their 10 populations they assumed a single true value of *δ* that is roughly applicable throughout the entire area occupied by the population. Thus, if investigators deployed traps in different patterns within the same population, while following the rules for estimating *S* [2], the resulting estimates of *δ* should be the same, regardless of the trap distribution adopted–subject only to experimental errors in estimating *b* and *N*_*e*_. We investigate whether this is true, using data from studies carried out in Tanzania and Uganda [2,7,11].

For the Tanzania study, using *G*. *pallidipes*, the original report states that sampling of tsetse was carried out using two traps at each of seven sites [7]. Because GPS coordinates were apparently not available for these traps, de Meeûs et al. analysed the data imagining that only one trap was used at each site. For such analyses they state: “when only one trap was available per site or when the GPS coordinates of corresponding traps (one subsample) were not available, we computed *S* = π(*D*_*min*_/2) ^2^ where *D*_*min*_ is the distance between the two closest sites taken as the distance between the centers of two neighboring subpopulations” [2].

However, there were actually two traps at each site, not one [7]. Suppose that GPS coordinates were available for the traps. Then it would be logical to employ the alternative definition for estimating *S*: “For all analyses, when more than one trap was available in a site, the surface area of the site was computed as *S* = π(*D*_*max*_) ^2^ where *D*_*max*_ is the distance between the two most distant traps in a given site, taken as the radius of the corresponding subpopulation.” [2]. We calculate, and compare, the values of $\hat{\delta }$ that result from the two different values of $\hat{S}$.

In the study of *G*. *fuscipes fuscipes* in Uganda, six traps were deployed at each of 42 sites, spread across an area of about 4000 km^2^ [11]. Traps at each site were separated by a distance of at least 100 m. For this trap spacing, a value of $\hat{S}$ = 0.02 km^2^ was calculated, using $\hat{S}$ = π(*D*_*max*_)^2^ (see above). Using finite estimates of ${\hat{N}}_{e}$, from 30 of the 42 sites, Opiro et al. calculate an arithmetic mean of ${\hat{N}}_{e}$ = 425 flies for the effective population (Fig A of S3 Text) [11]. The other 12 sites in the Opiro et al. study did not provide finite estimates of ${\hat{N}}_{e}$. Similarly, they estimated $\hat{b}$ = 0.0202 using information on genetic and geographic distances between all available sites. Using the above estimates for $\hat{S},\;\hat{b}$ and ${\hat{N}}_{e}$, de Meeûs et al. calculated $\hat{\delta }$ = 27 m per generation, the lowest among all of the 10 studies cited, and the one where the estimated effective population density, ${\hat{D}}_{e}$, was the second highest [2].

We carried out a “thought experiment” to investigate how the $\hat{S},\;{\hat{D}}_{e}$ and $\hat{\delta }$ estimates are affected by changes in trap deployment patterns. This was a simulation exercise, based solely on the data from the Uganda study and the NDDD protocol [2,11]. We imagined that the study was replicated 10 times, with different trap placements for each replicate, giving rise to different values of $\hat{S}$. We stipulated only that the traps were always distributed throughout the roughly 4000 km^2^ in the study area [11]. See S3 Text for full details of the analytic procedure, and Table B in S3 Table for the Excel file in which the simulations were executed – and where the reader can make repeat runs of the procedure.

## Results

### Analysis of relationships observed in the de Meeûs et al. study [2]

**Fig 1:**
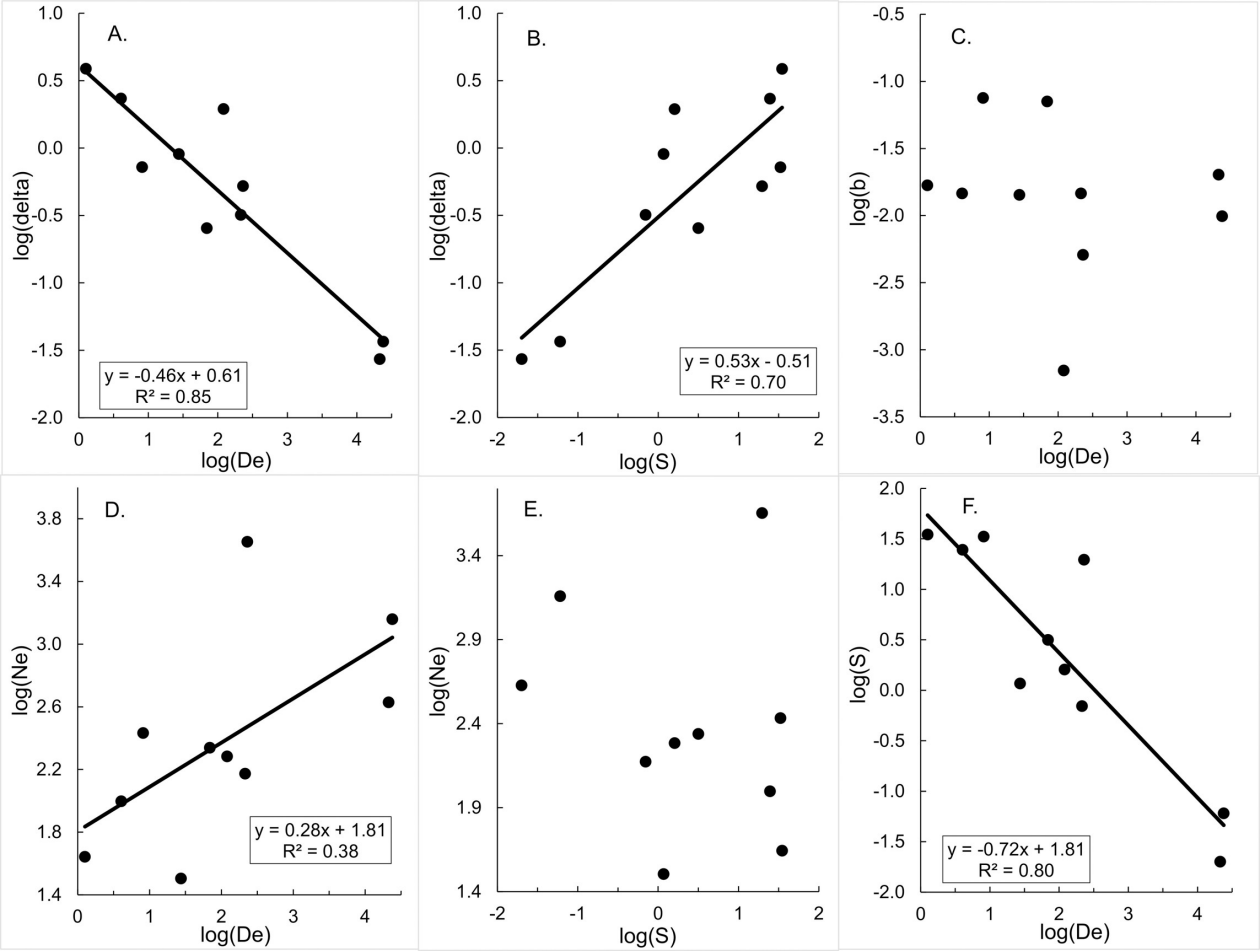
Relationships between various parameters in Eq (1) for data published in [2]. A. Predicted dispersal distance (log($\hat{\delta }$)) *vs* effective population density (log(${\hat{D}}_{e}$)). B. Predicted dispersal distance (log($\hat{\delta }$)) *vs* surface area (log($\hat{S}$)) occupied by the effective population. C. Regression coefficient (log(*b*)) *vs* effective population density (log(${\hat{D}}_{e}$)). D. Effective population size (log(${\hat{N}}_{e}$)) *vs* effective population density (log(${\hat{D}}_{e}$)). E. Effective population size (log(${\hat{N}}_{e}$)) *vs* surface area (log($\hat{S}$)) occupied by the effective population. F. Surface area (log($\hat{S}$)) occupied by the effective population *vs* effective population density (log(${\hat{D}}_{e}$)).

The primary observation of de Meeûs et al. was a strong linear correlation (*R*^2^ = 0.85; *P*<0.01) between log ($\hat{\delta }$), the dispersal per generation, and log (${\hat{D}}_{e}$), the effective population density, for the 10 populations studied, with a slope close to -0.5 (Fig 1A) [2]. Their results also showed a strong negative correlation (*R*^2^ = 0.86; *P*<0.02) between their predicted values of log($\hat{\delta }$) and the logs of the estimated census population densities (${\hat{D}}_{c}$ = ${\hat{N}}_{c}/\hat{S}$), with ${\hat{N}}_{c}$ – the census population – estimated from trap catches. These relationships form the central structure of the argument for NDDD. Notice that, in their data, log($\hat{b}$), the slope of the linear regression of genetic distance against log-transformed geographic distance, is not significantly correlated with log(${\hat{D}}_{e}$) (Fig 1C; *P* > 0.05). Similarly, log(${\hat{N}}_{e}$), the effective population size, is weakly correlated with log(${\hat{D}}_{e}$) (Fig 1D), and not significantly correlated with log($\hat{S}$), the area estimated to be occupied by *N*_*e*_ (Fig 1E). Surprisingly, log($\hat{\delta }$) is strongly correlated with log($\hat{S}$) (Fig 1B), and log($\hat{S}$) is strongly negatively correlated with log(${\hat{D}}_{e}$) (Fig 1F). The following sections clarify the origins of these counter-intuitive results.

### How measurement errors generate a false signal of NDDD with a slope of -0.5

Eq (1) is a rearrangement of the original derived by Rousset [1] to describe the value of *b* that would arise in a population as a result of given values of *D*_*e*_ and *δ*, where *D*_*e*_ = *N*_*e*_/*S* and *δ* is a distance measure somewhat different from that used in [2]. If the parameters in this equation could be measured without error, Eq (1) could indeed be a valid tool for obtaining a value for $\hat{\delta }$ based on estimates of ${\hat{D}}_{e}$ and $\hat{b}$, and then correlating $\hat{\delta }$ with ${\hat{D}}_{e}$. But that is not the case where *errors* occur in the estimate ${\hat{D}}_{e}$, whether these errors occur randomly or otherwise. When Eq (1) is used to calculate $\hat{\delta }$ from ${\hat{D}}_{e}$ the error in ${\hat{D}}_{e}$ will propagate to $\hat{\delta }$. This means that any overestimate of ${\hat{D}}_{e}$ will lead to an underestimate of $\hat{\delta }$, and vice-versa. If ${\hat{D}}_{e}$ and $\hat{\delta }$ are then plotted against each other, the result is the error in ${\hat{D}}_{e}$ being plotted against itself. If the error in ${\hat{D}}_{e}$ is large enough – and we will show later that errors can be >1000-fold – this autocorrelation will overwhelm any true relationship between *D*_*e*_ and *δ*. As the error in ${\hat{D}}_{e}$ increases, $\hat{b}$ approaches independence from ${\hat{D}}_{e}$, and d(ln(*δ*))/d(ln(*D*_*e*_)) – *i*.*e*., the rate of change of ln(*δ*) with changes in ln(*D*_*e*_) – can then be calculated from Eq (1) with *b* as a fixed parameter, which yields a slope of -0.5.

The complementary mathematical analysis in S2 Text confirms that, as long as estimation errors strongly dominate the values of ${\hat{D}}_{e}$, the relationship between log(${\hat{D}}_{e}$) and log($\hat{\delta }$) will appear to suggest the presence of NDDD, with a slope of -0.5, even in circumstances where the true situation is DID. We now use a simulation exercise to confirm this result.

#### A false signal of NDDD in a simulated population with assumed DID

**Fig 2:**
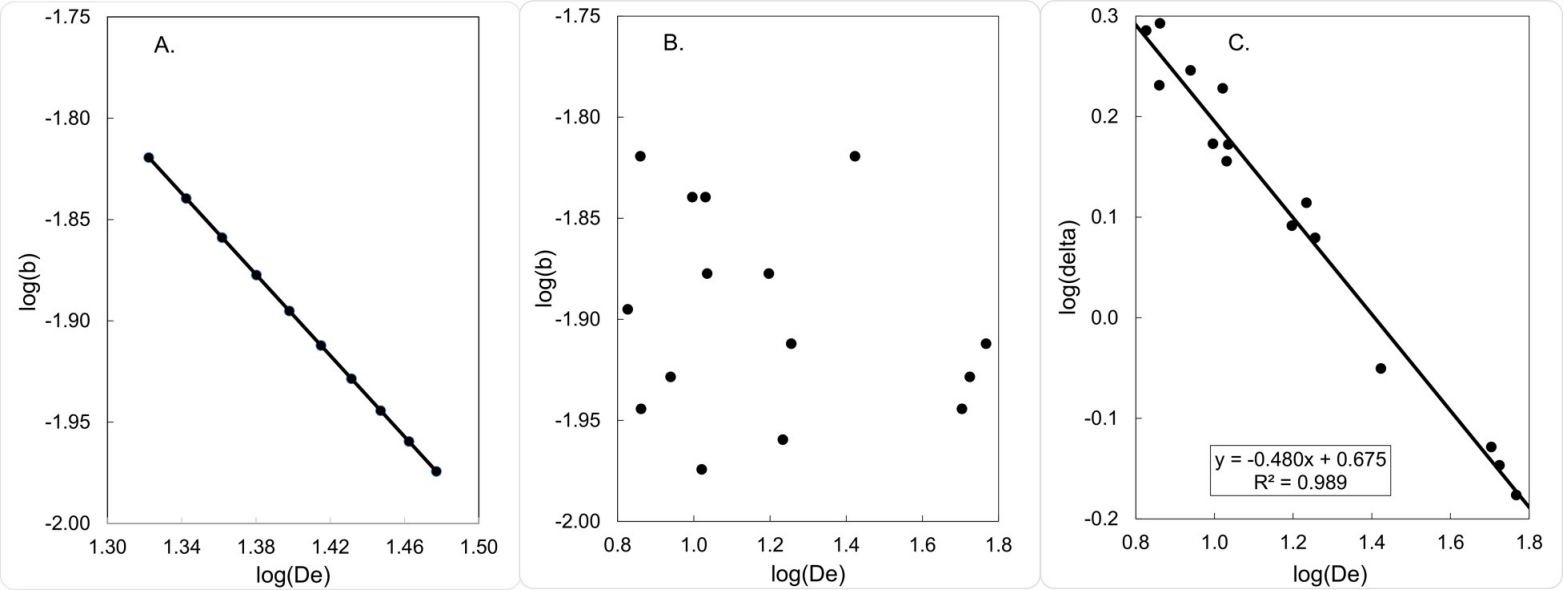
Simulation of a group of populations where the true dispersal rate (*δ*) is assumed fixed and independent of the true population density (*D*_*e*_). A. Relationship between log(*b*) and log(*D*_*e*_) when *D*_*e*_ is measured without error. B. Single realisation of the same relationship when *D*_*e*_ is measured with error. C. Single realisation of the relationship between log($\hat{\delta }$) and log(${\hat{D}}_{e}$) when *D*_*e*_ is measured with error. To generate further realisations of the process, and to explore the effect of changing the error in the estimate of *D*_*e*_, see Table A in S2 Table.

We tested the prediction that errors in measures of *D*_*e*_ lead naturally to the false signal of NDDD, using a simulated population where we specifically assumed DID. Thus, if *D*_*e*_ is measured without error, then calculated values of *b* declined as a power function of *D*_*e*_, to satisfy Eq **1** (Fig 2A). When there was error in *D*_*e*_, however, log(*b*) appeared uncorrelated with log(*D*_*e*_) (Fig 2B) and there was a negative correlation between log(${\hat{D}}_{e}$) and log($\hat{\delta }$), with a slope tending towards -0.5 (Fig 2C). This approximated the situation observed in plots of real field data (cf Figs 1C and 2B, and Figs 1A and 2C).

Notice that Fig 2 provides a single realisation of a random process. The reader can vary the input values in Table A in S2 Table to observe the consequences for the slope of log($\hat{\delta }$) against log(${\hat{D}}_{e}$). Since the simulation is stochastic, the slope changes with each realisation of the process but, for fold-error greater than around 1.2, the slope is invariably less than zero, as illustrated in Fig A in S2 Table. That is to say, the population appears to exhibit NDDD, despite the fact that it has been set up such that dispersal is actually independent of population density. Since ${\hat{D}}_{e}$ = ${\hat{N}}_{e}/\hat{S}$, errors in $\hat{S}$ can lead to errors in ${\hat{D}}_{e}$. We now show that there are indeed serious errors in the estimation of *S*: these errors are central to the false signal of NDDD.

#### The importance of $\hat{S}$ in determining the value of $\hat{\delta }$

Given that $\hat{S}$ represents an estimate of the area covered by a collection traps, there is no biological reason to expect the high correlations that de Meeûs et al. [2] found between log(${\hat{D}}_{e}$) and log($\hat{S}$) and between log($\hat{\delta }$) and log($\hat{S}$) (Fig 1A and 1B). The fact that strong correlations were found, led us to explore whether the variation that de Meeûs et al. observed in ${\hat{D}}_{e}$ was mostly driven by changes in the estimated area ($\hat{S}$) covered by traps, rather than by true changes in *D*_*e*_ [2]. As described in the Methods section, we investigated the importance of *S* in determining the value of δ predicted by Eq (2), relative to contributions from other terms. These analyses yielded the following percentages for the three predictors: log(${\hat{N}}_{e}$) = 25%, log($\hat{S}$) = 65%, and log($\hat{b}$) = 10%. Thus, the variation in log(*Ŝ*) explained almost twice as much of the variation in log($\hat{\delta }$) as did the other two variables combined.

#### The importance of errors in $\hat{S}$ in creating a false signal of NDDD

The central importance of *S* in accounting for variation in the dispersal rate (*δ*) implies that errors in $\hat{S}$ will be crucial in determining errors in ${\hat{D}}_{e}$ and thus $\hat{\delta }$. This leads inevitably to the main problem with the NDDD protocol, embodied in the statement: “The average surface (*S*) occupied by a subpopulation can be computed as the surface area occupied by the different traps used in a given survey site” [2]. In fact, the relationship between the true area (*S*) covered by a biological subpopulation and the area ($\hat{S}$) estimated to be covered by a set of traps, remains unknown. Methods for estimating *N*_*e*_ assume that there is no genetic structure within a sub-population [24], meaning that there should be no systematic genetic distinction between flies caught in different traps. Thus, *N*_*e*_ can be estimated by sampling from any area smaller than the geographic range over which the sub-population can be said to be well mixed genetically.

**Fig 3:**
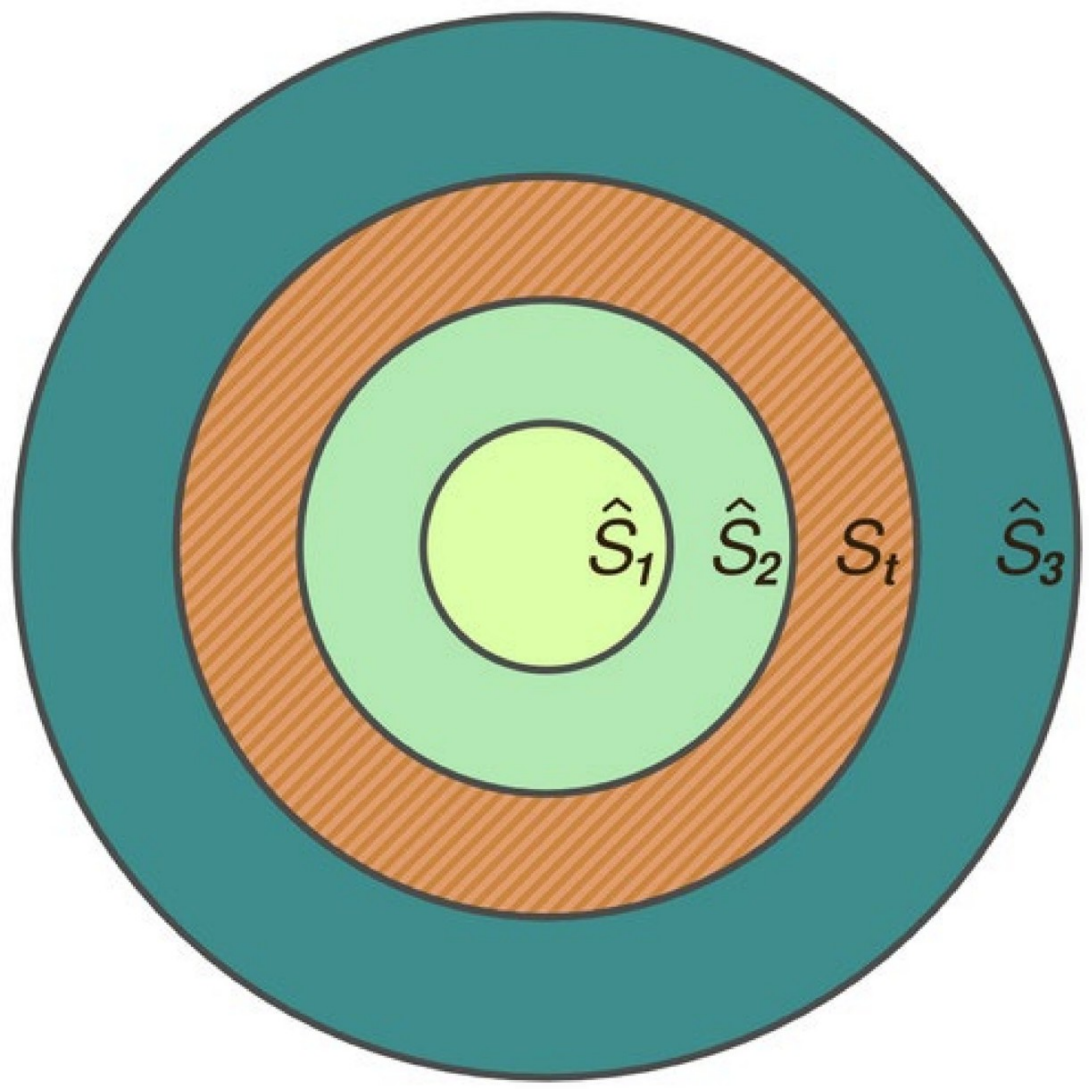
Sampled and true subpopulation areas. Illustration of three different possible sampling areas (${\hat{S}}_{1}–{\hat{S}}_{3}$) relative to the true area (*S*_*t*_) covered by a subpopulation.

Fig 3 shows three different possibilities for the trap-sampling areas (${\hat{S}}_{1}$ - ${\hat{S}}_{3}$) in relation to the true area (*S*_*t*_) occupied by a hypothetical, well-mixed subpopulation. Sampling flies from within either ${\hat{S}}_{1}$ or ${\hat{S}}_{2}$ will produce the same expected value of the estimate ${\hat{N}}_{e}$, of the true value of *N*_*e*_, but will use different values of $\hat{S}$, which is the denominator for calculating ${\hat{D}}_{e}$. Thus, the estimated population density will vary according to the area covered by the traps, introducing error into the ${\hat{D}}_{e}$ estimates.

If ${\hat{S}}_{3}$ is used (covering an area larger than the size of what can be considered a well-mixed subpopulation), then the assumptions underlying the estimates of ${\hat{N}}_{e}$ will be violated, and the estimates of ${\hat{N}}_{e}$ will be flawed, so introducing yet more error to ${\hat{D}}_{e}$. The extent to which these erroneous estimates of ${\hat{N}}_{e}$ will scale with $\hat{S}$ – as it grows above the size of *S* – is not entirely clear, but previous work suggests that it will not scale linearly [25] and will thus continue to produce incorrect values of ${\hat{D}}_{e}$ that are a function of trap placement. Correct values of ${\hat{D}}_{e}$ can be obtained only in the special case where *S* = *S*_*t*_. We will now show that, for the data used in the NDDD protocol, variation in ${\hat{D}}_{e}$ is due largely to the choice of trap placement, and the way in which those placements are interpreted, rather than to true biological variation in *N*_*e*_ [2].

### Simulations illustrating false signals of NDDD in field studies

#### Study on *G*. *pallidipes* in Tanzania [7]

These data were originally analysed in the NDDD protocol as if only one trap was used at each site, with a value of *D*_*min*_ ≈ 6.7 km, so that $\hat{S}$ = π(*D*_*min*_/2) ^2^ = 34.87 km^2^ [2]. Using the resulting estimated values of $\hat{b}$ = 0.0168 and ${\hat{N}}_{e}$ = 44, the authors calculated a value of $\hat{\delta }$ = 3875 m per generation from Eq (1), the highest estimate in any of the studies they cited [2]. As we noted in the Methods section, however, there were actually two traps at each site, not one. Although GPS coordinates were lacking for these traps, the distance between them was known (100 m). Using the definition appropriate for this situation gives *D*_*max*_ = 0.1 km, resulting in a value of $\hat{S}$ = π(*D*_*max*_)^2^ ≈ 0.031 km^2^, differing from the published estimate of $\hat{S}$ by a factor of more than 1000 [2].

Notice that, whichever concept of trap spacing was used, the same flies would have provided the genetic and geographic information employed in the published estimates of $\hat{b}$ and ${\hat{N}}_{e}$ [2] which would thus have been identical in each case. Accordingly, the revised estimates of effective population density is ${\hat{D}}_{e}$ ≈ 44/0.031 = 1401 tsetse per km^2^, and dispersal distance is *δ*≈$\sqrt{0.031}/\sqrt{\pi \times 0.0168\times 44}$ = 116 m, differing from the published estimate of δ by a factor of 33.4 [2]. We emphasise that either of the methods for estimating *S* presented by De Meeûs et al. [2] could reasonably be used for this dataset, and that the simple choice of which to apply caused this large difference in outcome.

This analysis suggests that the values of $\hat{\delta }$ obtained in all of the studies used by de Meeûs et al. [2], were strongly dependent on the spacings of the traps used to sample the population, and also on the decision about how to interpret those spacings. Alternatively, if it is objected that – by regarding the trap deployment as either one, or two, traps per site – the procedure is measuring the movement rates in two different subpopulations, we would be forced to conclude that values of *δ* could differ by orders of magnitude between subpopulations of the same population. Either scenario is sufficient to undermine the basis of the NDDD protocol [2].

#### Study on *G*. *f*. *fuscipes* in Uganda

Analysis of data from the Opiro et al. study of *G*. *f*. *fuscipes* in Uganda [11], casts further doubt on the NDDD hypothesis. As described in the Methods and in S3 Text and S3 Table we estimate values of ${\hat{D}}_{e}$ and $\hat{\delta }$ for simulated studies where 10 different trap deployment patterns were used throughout the roughly 4000 km^2^ of the study area [11]. If, as clearly assumed by the NDDD analysis [2], the expected value of δ is constant across the whole study area [11], then the analysis in each of our 10 estimation procedures, above, should provide roughly the same value of $\hat{\delta }$ – allowing for errors in the measurement of *b* and *N*_*e*_. That is to say, $\hat{\delta }$ should be independent of patterns of trap deployment. In particular, if log($\hat{\delta }$) is plotted against log(${\hat{D}}_{e}$) or against log($\hat{S}$), the results should approximate horizontal lines.

**Fig 4:**
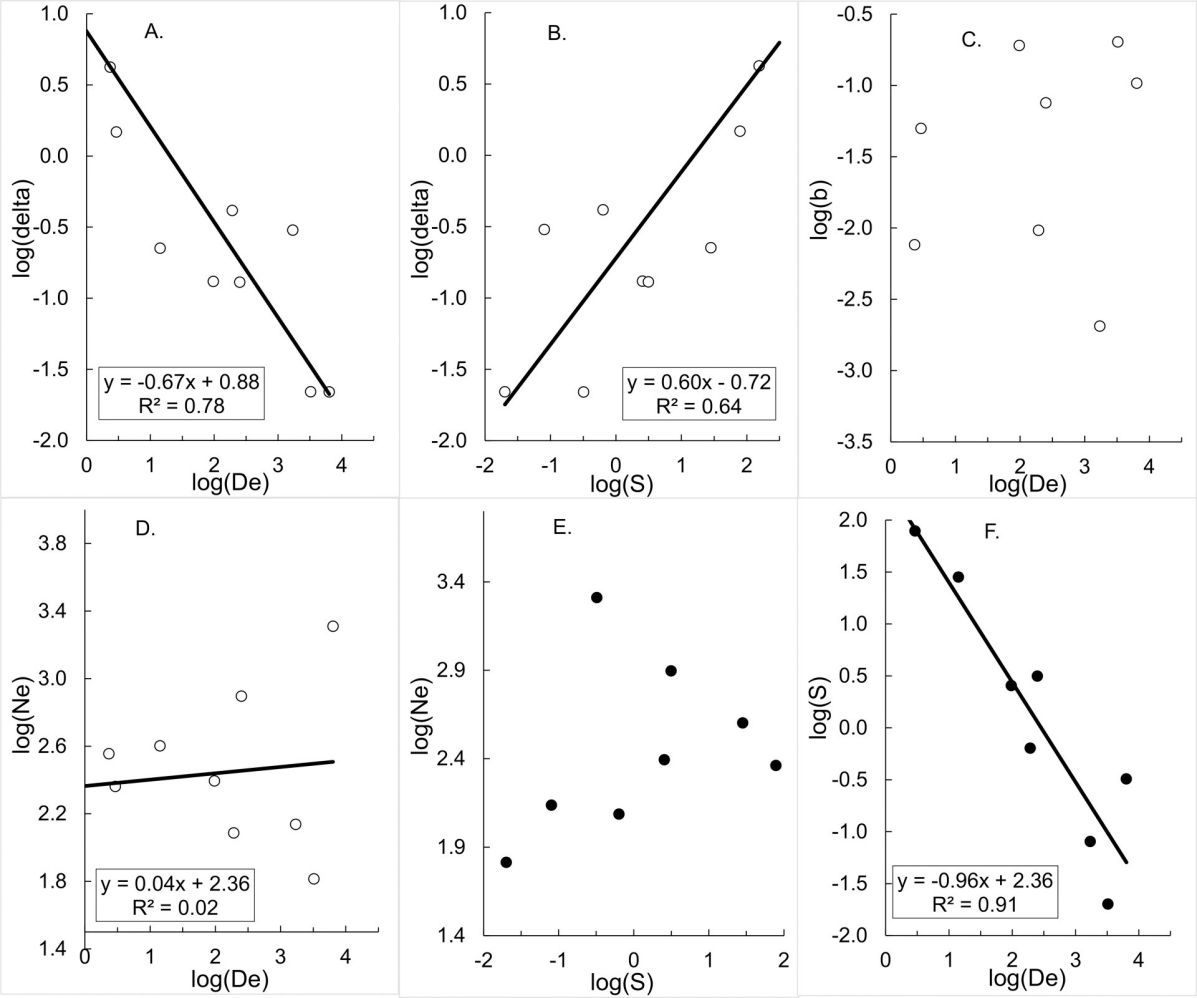
Simulations illustrating the false signal of NDDD in a field study. Parameter estimates generated for 10 different trap displacement choices applied to the Opiro et al. study on *G*. *f*. *fuscipes* in Uganda [11]. A. Dispersal distance (log($\hat{\delta }$)) *vs* effective population density (log(${\hat{D}}_{e}$)). B. Dispersal distance (log($\hat{\delta }$)) *vs* surface area (log($\hat{S}$)) occupied by the effective population. C. Regression coefficient (log(*b*)) *vs* effective population density (log(${\hat{D}}_{e}$)). D. Effective population size (log(${\hat{N}}_{e}$)) *vs* effective population density (log(${\hat{D}}_{e}$)). E. Effective population size (log(${\hat{N}}_{e}$)) *vs* surface area (log($\hat{S}$)) occupied by the effective population. F. Surface area (log($\hat{S}$)) occupied by the effective population *vs* effective population density (log(${\hat{D}}_{e}$)).

The reality is markedly different. The results of a single realisation of the simulation procedure are shown in Fig 4, from which it is seen that the simulation essentially reflects all of the properties of the NDDD picture provided in Fig 1. In particular we see that: log($\hat{\delta }$) is strongly correlated with log(${\hat{D}}_{e}$), with a slope around -0.5 (Fig 4A); log($\hat{\delta }$) is strongly correlated with log($\hat{S}$), with a slope around 0.5 (Fig 4B); log($\hat{b}$) and log(${\hat{D}}_{e}$) are poorly correlated (Fig 4C). Moreover, log($\hat{b}$) and log(${\hat{D}}_{e}$) are poorly correlated (Fig 4D); log(${\hat{N}}_{e}$) and log(${\hat{D}}_{e}$) are positively, but rather weakly, correlated (Fig 4E), and log($\hat{S}$) declines linearly with increasing log(${\hat{D}}_{e}$) (Fig 4F). The reader is invited to use the algorithm in Table B in S3 Table to make serial iterations of the stochastic procedure – with each iteration using a different randomly generated error for log($\hat{b}$) and log(${\hat{N}}_{e}$).

Notice that we make no assumption about the true underlying nature of dispersal: it could be NDDD, DID or PDDD (positive density-dependent dispersal). Regardless of this, however, the output always strongly resembles NDDD. Moreover, while we have used only one of the ten studies involved in generating the NDDD hypothesis, the result is entirely general and the same problem will arise in the analysis of any of the studies cited [2,11]. Notice also that, if *b* and *N*_*e*_ are measured without error, the outcome still gives the appearance of NDDD (Table B and Fig A in S3 Table). That is to say, the false signal of NDDD is entirely due to the fundamentally erroneous assumption that $\hat{S}$, as measured by the distribution of traps used in the sampling procedure, provides a good estimate of the true area (*S*) occupied by the tsetse population under study.

The foregoing analyses show that, depending on trap placement and the choice of how $\hat{S}$ is calculated from such placement, the Tanzanian and Ugandan studies can both be made to reflect either an extremely high effective population density and low dispersal rate, or completely the reverse [7,11]. Clearly, in each study, these scenarios cannot both be correct. Indeed, both are almost certainly incorrect because, as explained above, $\hat{S}$ is virtually never equal to the true area (*S*) occupied by a subpopulation.

## Discussion

The primary goal of this paper was to assess the evidence relating to the notion of NDDD in tsetse. If support had been found for the idea, then the very general nature of the model on which NDDD was based [1], might suggest that the phenomenon could occur in other creatures, including other insect vectors of disease. Our simulations and arguments show, however, that there are serious flaws in the arguments that led to the notion of NDDD in tsetse [2]. These flaws can be summarised by the following two points. First, the use of Eq 1 to calculate $\hat{\delta }$ from measurements of ${\hat{D}}_{e}$ is biased towards finding a spurious correlation between $\hat{\delta }$ and ${\hat{D}}_{e}$, whenever there is error in the measurement of ${\hat{D}}_{e}$. Since population genetic parameter estimates will always contain some degree of error, this alone is concerning. Second, the methods used for estimating $\hat{S}$ introduce very large errors into the estimates of ${\hat{D}}_{e}$ = ${\hat{N}}_{e}/\hat{S}$. As the error in ${\hat{D}}_{e}$ becomes large, the predicted log slope of the spurious correlation with $\hat{\delta }$ will tend to -0.5. Hence, the procedure is inherently biased towards finding NDDD as marked as that actually observed by de Meeûs et al. even when dispersal is independent of density [2]. By far the largest errors in measurement appear to be in the estimate $\hat{S}$, and hence ${\hat{D}}_{e}$, and these stem from the notion that the true areas inhabited by the various subpopulations under study are determined by the distance between traps. That is the root error, since inter-trap distances are not features of tsetse populations. Instead, they are strongly constrained by logistical issues, such as the numbers of available paths and traps, the mode of transport employed, the purpose of the trapping study and the whims of the researcher.

The issues raised in the Results section provide good evidence, of themselves, to reject the NDDD hypothesis for tsetse. However, various other problems, explored in detail in S4 Text, cast further doubt on the credibility of the hypothesis and its associated arguments. Many of these problems relate to the interpretation of trap catches. Thus, errors in census population estimates result from an erroneous understanding of the relationship between trap placement and expected tsetse catch. This is exacerbated through failure to adjust for variations in trapping intensity, trap performance, and in capture probabilities between geographical situations and between tsetse species. Such problems lead to serious errors in estimates of census populations, and a consequent absence of the expected high correlation between the estimates of census and effective population numbers, ${\hat{N}}_{c}$ and ${\hat{N}}_{e}$ [2]. We also point out that there is no credible suggestion for any mechanism by which NDDD might have evolved and, contrary to claims in [2], no support in the literature for NDDD in tsetse. Indeed, available published evidence seems to suggest that PDDD is more likely than NDDD. Finally, we note that – even if we take the correlations in Fig 1 at face value – de Meeûs et al. [2] drew no distinction between correlation and causation, and did not consider the possibilities of reverse causality or confounding.

These considerations all give background support for our present results with tsetse and offer general warnings about problems that could occur if the NDDD protocol developed for tsetse were used with other creatures. As an important example, if trapping exercises, and genetic analyses, were carried out on mosquitoes – without due regard to the problems of correctly estimating the area *S* occupied by a subpopulation – then we can be sure that the same signal of NDDD would result, regardless of the true nature of dispersal in the mosquitoes. This could have serious consequences for decisions regarding vector control in support of efforts to combat the many human diseases that mosquitoes transmit.

Although NDDD was initially offered as a strongly supported hypothesis in need of testing, some of the co-authors are already treating the hypothesis as established fact. It is stated, for example, that: “through genetic studies, De Meeûs et al. (2019) have shown that a strong negative density-dependent dispersal occurs after control operations” [26], incorrectly implying that dispersal had been measured before and after control, so giving unwarranted weight to NDDD and the claimed risks that such dispersal poses for tsetse control. Such statements, taken with the warning that NDDD will unleash massive invasion from neighbouring untreated areas, must be seen as having potentially serious impacts on disease control policy. It is for that reason that we have felt it essential to expose so fully the errors involved in the NDDD hypothesis.

The danger in the over-hasty transformation of an intriguing hypothesis into an established "fact" is that caution is easily abandoned, and that support for the hypothesis can be seen where none occurs. For example, a claim for the existence of NDDD, which involves believing that sparse populations evolve to disperse especially widely, has been made while also stressing the contradictory proposition that such dispersal is markedly disadvantageous to the survival of sparse populations [26]. Similar problems have arisen in the history of tsetse control. For example, the hypothesis that tsetse have open "feeding grounds" and well-wooded "homes" [27–29] encouraged the unnecessary destruction of large areas of natural woodland in the name of tsetse control [30].

Nonetheless, it would clearly be beneficial if genetic analysis could be used to provide useful indications of the dispersal rate of tsetse, or indeed of any other creature. As we have seen, a central problem is that of providing accurate and meaningful estimates of population density, which requires in turn good estimates of the area (*S*) occupied by a population. The problem of providing estimates of absolute numbers, and densities, of tsetse populations has taxed workers for nearly a century–since C.H.N. Jackson pioneered the use of mark-recapture in a remarkable series of population studies with tsetse [27–29,31] – and we still do not have good solutions. The best estimates of census population numbers and densities (*N*_*c*_ and *D*_*c*_) have involved mark-recapture exercises applied to populations on an island of known area, and closed to immigration and migration [32,33].

When such exercises are carried out on open populations, the results are much more difficult to interpret. For example, estimates of census numbers of *G*. *m*. *morsitans* and *G*. *pallidipes* near Rekomitjie Research Station, in Zimbabwe, varied by between 3 and 12-fold depending on whether numbers were estimated using mark-recapture, or a dynamic system that modelled rates of population change due to in- and out-migration as well as death and trapping [34,35]. Discrepancies were biggest for *G*. *pallidipes*, the larger and more mobile species, underlining the confusing effect of varied rates of fly dispersal. However, the problem lies more in estimating the area (*S*) occupied by the study population than in estimating the census numbers themselves. Moreover, while the effective population numbers (*N*_*e*_) can be estimated from genetic data [36,37] there is still the problem of estimating *S* and, as we have seen in this paper, that difficulty lies at the heart of the efforts made to estimate dispersal rates from the analyses of genetic data [2]. We cannot ourselves suggest a solution to this problem but feel that it is important to highlight it for further consideration.

## Supporting information

S1 Table Data used to create Fig 1 of de Meeûs et al (2019). **Table A in S1 Table.** Data kindly provided by Dr Thierry de Meeûs.
(XLSX)

S2 TableFalse signal of NDDD in a simulated population with assumed DID.**Table A in S2 Table.** Simulation of situation where dispersal rates are independent of population density but where *D*_*e*_ is measured with error. **Fig A in S2 Table.** Simulation of a group of populations where the true dispersal rate (δ) is independent of the true population density (*D*_*e*_). A. Relationship between log($\hat{b}$) and log(${\hat{D}}_{e}$) when De is measured without error. B. Single realisation of the same relationship when *D*_*e*_ is measured with error. C. Single realisation of the relationship between log($\hat{\delta }$) and log(${\hat{D}}_{e}$) when De is measured with error.(XLSX)

S3 TableCalculations for “Thought Experiment”.**Data from Opiro (2017). Table A in S3 Table.** Data kindly provided by Dr Thierry de Meeûs. **Table B in S3 Table.** Simulations used to estimate dispersal parameters where there may be errors of estimation for *b*, *S* and *N*_*e*_. **Fig A in S3 Table. Simulations illustrating the false signal of NDDD in a field study.** Parameter estimates generated for 10 different trap displacement choices applied to the Opiro et al. (2017) study of *G*. *f*. *fuscipes* in Uganda. A. Dispersal distances (log($\hat{\delta }$)) *vs* effective population density (log(${\hat{D}}_{e}$)). B. Dispersal distance (log($\hat{\delta }$)) *vs* surface area (log($\hat{S}$)) occupied by the effective population. C. Regression coefficient (log($\hat{b}$)) *vs* effective population density (log(${\hat{D}}_{e}$)). D. Effective population size (log(${\hat{N}}_{e}$)) *vs* effective population density (log(${\hat{D}}_{e}$)). E. Effective population size (log(${\hat{N}}_{e}$)) *vs* surface area (log($\hat{S}$)) occupied by the effective population. F. Surface area (log($\hat{S}$)) occupied by the effective population *vs* effective population density (log(${\hat{D}}_{e}$)). **Fig B in S3 Table.** Simulations using Opiro et al (2017) data, with or without error. Row A: Results of de Meeûs et al. (2019) for comparison. Row B: Recalculation of Opiro et al (2017) results for varying trap displacements, with error. Row C: Recalculation of Opiro et al (2017) results for varying trap displacements without error.(XLSX)

S1 Text Estimates of population densities and dispersal per generation. **Table A in S1 Text.** Data used by de Meeûs et al. (2019a) in the production of their Fig 1.
(DOCX)

S2 Text Mathematics of the “errors-in-D_e_” argument. (DOCX)

S3 TextProcedure for “Thought Experiment”.**Data from Opiro (2017). Fig A in S3 Text. Distribution of estimates of** ${\hat{N}}_{e}$ **from the Opiro et al. (2017) study**. Of the 42 study sites, only 30 provided finite estimates of ${\hat{N}}_{e}$.(DOCX)

S4 TextMiscellaneous problems relating to the NDDD hypothesis.A. Errors involved in the estimation of *b*. B. Errors involved in the estimation of *N*_*c*_, the true census density. C. Absence of correlation between ${\hat{N}}_{c}$ and ${\hat{N}}_{e}$. D. Failure to allow for intensity and duration of trapping, differences in performance between traps, between species and between geographical/ecological regions E. Contradictory evidence from trap catches. F. Inappropriate pooling of data for different situations. G. Unsupported claims of effects of NDDD reinvasion dynamics. H. No field evidence for larviposition pheromone in tsetse. I. Absence of any suggestion for a mechanism by which NDDD might have evolved. J. Errors in claimed support for NDDD. K. PDDD more likely than NDDD. L. Confusion between correlation and causation; possible reverse causality and confounding. **Fig A in S4 Text.** Data and fitted IBD regression for *G*. *tachinoides* study in Ghana. Row 2, Table A reports regression statistics (Adam et al., 2014). **Table A in S4 Text.** Significance testing of IBD regression slope, *b*. De Meeûs et al. (2019a) supplied values of *b* and its CI, estimated via bootstrap-over-loci (BOL) for 7 studies, and also supplied Mantel test results for 3 studies. We calculated jackknife estimates of *b* and its 95% CI for 7 of the 10 studies, using genetic distances based on all available loci. We estimated squared Pearson correlations (*R*^*2*^) from all available genetic and geographic distances, with the full subset of loci included in genetic distances. **Fig B in S4 Text.** Surface area occupied by a subpopulation, as estimated using the NDDD protocol. A. More than one trap deployed at a site. In the example shown (*cf* (Opiro et al., 2017) five traps are spaced at equal intervals on the circumference of a circle of radius *r* units, with a further trap at the centre of the circle. The protocol calculate the area of the site as $\hat{S}$ = π(*D*_*max*_)^2^, where *D*_*max*_ is the distance between the two most distant traps in a given site, taken as the radius of the corresponding subpopulation. With this pattern, *D*_*max*_ ≈ 1.9 *r*. B. Two trapping sites with one trap deployed at each site. For this scenario, the surface area occupied by the sub-population sampled is calculated from *S* = π(*D*_*min*_/2)^2^, where *D*_*min*_ is the distance the distance between the centres of two neighbouring subpopulations and thus as the average diameter of a subpopulation. **Fig C in S4 Text. Effective and census population numbers and densities using data from de Meeûs et al. (2019a).** Plots of: A. census population size (${\hat{N}}_{c}$) against effective population size (${\hat{N}}_{e}$); B. census population density (${\hat{D}}_{c}$) against effective population density (${\hat{D}}_{e}$). All data transformed by taking logs to the base 10.(DOCX)

## References

[1] Rousset F. Genetic differentiation and estimation of gene flow from F-statistics under isolation by distance. Genetics. 1997;145:1219–28. 9093870 10.1093/genetics/145.4.1219 PMC1207888

[2] De Meeûs T, Ravel S, Solano P, Bouyer J. Negative Density-dependent Dispersal in Tsetse Flies: A Risk for Control Campaigns? Trends Parasitol. 2019;35(8):615–21. 10.1016/j.pt.2019.05.007 31201131

[3] Adam Y, Bouyer J, Dayo GK, Mahama CI, Vreysen MJB, Cecchi G, et al. Genetic comparison of Glossina tachinoides populations in three river basins of the Upper West Region of Ghana and implications for tsetse control. Infect Genet Evol. 2014;28:588–95. 10.1016/j.meegid.2014.03.023 24709401

[4] Hyseni C, Kato AB, Okedi LM, Masembe C, Ouma JO, Aksoy S, et al. The population structure of Glossina fuscipes fuscipes in the Lake Victoria basin in Uganda: Implications for vector control. Parasites and Vectors. 2012;5(1):1–14. 10.1186/1756-3305-5-1 23036153 PMC3522534

[5] Koné N, Bouyer J, Ravel S, Vreysen MJB, Domagni KT, Causse S, et al. Contrasting population structures of two vectors of African Trypanosomoses in Burkina Faso: Consequences for control. PLoS Negl Trop Dis. 2011;5(6):1–10. 10.1371/journal.pntd.0001217 21738812 PMC3125141

[6] Manangwa O, Nkwengulila G, Ouma JO, Mramba F, Malele I, DIon K, et al. Genetic diversity of Glossina fuscipes fuscipes along the shores of Lake Victoria in Tanzania and Kenya: Implications for management. Parasites and Vectors. 2017;10(1):4–11. 10.1186/s13071-016-1940-4 28558831 PMC5450392

[7] Manangwa O, De Meeûs T, Grébaut P, Ségard A, Byamungu M, Ravel S. Detecting Wahlund effects together with amplification problems: Cryptic species, null alleles and short allele dominance in Glossina pallidipes populations from Tanzania. Mol Ecol Resour. 2019;19(3):757–72. 10.1111/1755-0998.12989 30615304

[8] Melachio T, Simo G, Ravel S, De Meeûs T, Causse S, Solano P, et al. Population genetics of Glossina palpalis palpalis from central African sleeping sickness foci. Parasites and Vectors. 2011;4(1):1–8. 10.1186/1756-3305-4-140 21767402 PMC3162924

[9] Mélachio TTT, Njiokou F, Ravel S, Simo G, Solano P, De Meeûs T. Effect of sampling methods, effective population size and migration rate estimation in Glossina palpalis palpalis from Cameroon. Infect Genet Evol. 2015;33:150–7. 10.1016/j.meegid.2015.04.023 25917495

[10] Okeyo WA, Saarman NP, Mengual M, Dion K, Bateta R, Mireji PO, et al. Temporal genetic differentiation in Glossina pallidipes tsetse fly populations in Kenya. Parasites and Vectors. 2017;10(1):1–13. 10.1186/s13071-016-1943-1 29017572 PMC5635580

[11] Opiro R, Saarman NP, Echodu R, Opiyo EA, Dion K, Halyard A, et al. Genetic diversity and population structure of the tsetse fly Glossina fuscipes fuscipes (Diptera: Glossinidae) in Northern Uganda: Implications for vector control. PLoS Negl Trop Dis. 2017;11:1–29. 10.1371/journal.pntd.0005485 28453513 PMC5425221

[12] Hargrove JW. Tsetse eradication: sufficiency, necessity and desirability. Edinburgh, UK: DFID Animal Health Programme; 2003. 133 p.

[13] Feldmann U. The sterile insect technique as a component of area-wide integrated pest management. In: Maudlin I., Holmes P.H., Miles AM, editor. The Trypanosomiases. Wallingford, UK: CABI Publishing; 2004. p. 614.

[14] Bourn D, Grant I, Shaw A, Torr S. Cheap and safe tsetse control for livestock production and mixed farming in Africa. Asp Appl Biol. 2005;75:81.

[15] Shaw APM, Torr SJ, Waiswa C, Cecchi G, Wint GRW, Mattioli RC, et al. Estimating the costs of tsetse control options: An example for Uganda. Prev Vet Med. 2013;110(3–4):290–303. 10.1016/j.prevetmed.2012.12.014 23453892

[16] Shaw APM, Tirados I, Mangwiro CTN, Esterhuizen J. Costs of using “tiny targets” to control Glossina fuscipes fuscipes, a vector of gambiense sleeping sickness in Arua district of Uganda. PLoS Negl Trop Dis. 2015;9:1–19. 10.1371/journal.pntd.0003624 25811956 PMC4374750

[17] Shereni W, Neves L, Argilés R, Nyakupinda L, Cecchi G. An atlas of tsetse and animal African trypanosomiasis in Zimbabwe. Parasit Vectors. 2021;14(1):50. 10.1186/s13071-020-04555-8 33446276 PMC7807824

[18] De Meeûs T, Ravel S, Solano P, Bouyer J. Response to the comments of J. S. Lord. Trends Parasitol. 2019;35:742. 10.1016/j.pt.2019.07.009 31431328

[19] Hargrove J.W., Vale GA. Negative density-dependent dispersal in tsetse (Glossina spp): red flag or red herring? Med Vet Entomol. 2020;In press:1–12. 10.1111/mve.12466 32757252

[20] Charlesworth B. Fundamental concepts in genetics: Effective population size and patterns of molecular evolution and variation. Nat Rev Genet. 2009;10(3):195–205. 10.1038/nrg2526 19204717

[21] Chevan A, Sutherland M. Hierarchical partitioning. Am Stat. 1991;45(2):90–6.

[22] Mac Nally R. Regression and model-building in conservation biology, biogeography and ecology: The distinction between–and reconciliation of–‘predictive’ and ‘explanatory ‘ models. Biodivers Conserv. 2000;9:655–71.

[23] Murray K, Conner M. Methods to quantify variable importance: implications for the analysis of noisy ecological data. Ecology. 2009;90(2):348–55. 10.1890/07-1929.1 19323218

[24] Bartley D, Bagley M, Gall G, Bentley B. Use of Linkage Disequilibrium Data to Estimate Effective Size of Hatchery and Natural Fish Populations. Conserv Biol. 1992;6(3):365–75.

[25] Neel MC, Mckelvey K, Ryman N, Lloyd MW, Bull RS, Allendorf FW, et al. Estimation of effective population size in continuously distributed populations: there goes the neighborhood. Heredity (Edinb). 2013;111(May):189–99. 10.1038/hdy.2013.37 23652561 PMC3746818

[26] Gimonneau G, Ouedraogo R, Salou E, Rayaisse JB, Buatois B, Solano P, et al. Larviposition site selection mediated by volatile semiochemicals of larval origin in Glossina palpalis gambiensis. Ecol Entomol. 2020;

[27] Jackson CHN. The analysis of a tsetse fly population. Ann Eugen. 1941;10.:332–69. 10.1111/j.1469-1809.1947.tb02381.x 18863973

[28] Jackson CHN. The analysis of a tsetse fly population II. Ann Eugen. 1944;12:176–205. 10.1111/j.1469-1809.1947.tb02381.x 18863973

[29] Jackson CHN. The analysis of a tsetse fly population. III. Ann Eugen. 1948;14:91–108. 10.1111/j.1469-1809.1947.tb02381.x 18863973

[30] Ford J., Nash T.A.M., Welch JR. Control by clearing of vegetation. In: Mulligan HW, editor. The African Trypanosomiases. London: George Allen & Unwin, London; 1970. p. 543–63.

[31] Jackson CHN. An artificially isolated generation of tsetse Flies (Diptera). Bull Entomol Res. 1946;37:291–9. 10.1017/s0007485300022203 21000966

[32] Vale G., Hargrove JW, Cockbill GF, Phelps RJ(1986). Field trials of baits to control populations of Glossina morsitans morsitans Westwood and G. pallidipes Austen (Diptera: Glossinidae). Bull Entomol Res. 1986;76:179–93.

[33] Hargrove JW. Optimized simulation of the control of tsetse flies Glossina pallidipes and G. m. morsitans (Diptera: Glossinidae) using odour-baited targets in Zimbabwe. Bull Entomol Res. 2003;93(1). 10.1079/BER2002205 12593679

[34] Phelps RJ, Vale GA. Studies on populations of Glossina morsitans morsitans and G. pallidipes (Diptera: Glossinidae) in Rhodesia. J Appl Ecol. 1978;15(3):743.

[35] Hargrove JW. Discrepancies Between Estimates of Tsetse Fly Populations Using Mark- Recapture and Removal Trapping Techniques. J Appl Ecol. 1981;18(3):737–48.

[36] Shirk AJ, Cushman SA. Spatially-explicit estimation of Wright’s neighborhood size in continuous populations. Front Ecol Evol. 2014;2:1–12.

[37] Jasper M, Schmidt TL, Ahmad NW, Sinkins SP, Hoffmann AA. A genomic approach to inferring kinship reveals limited intergenerational dispersal in the yellow fever mosquito. Mol Ecol Resour. 2019;19(5):1254–64. 10.1111/1755-0998.13043 31125998 PMC6790672

