# Supplementary material for: Negative density-dependent dispersal in tsetse (Glossina spp): An artefact of inappropriate analysis
Source: PLoS Negl Trop Dis. 2021 Mar 25;15(3):e0009026. doi: 10.1371/journal.pntd.0009026 (PMC8023489; doi:10.1371/journal.pntd.0009026)
Supplement: S1 Text — Table A in S1 Text. Data used by de Meeûs et al. (2019a) in the production of their Fig 1. (DOCX) [file pntd.0009026.s004.docx]

**Negative density-dependent dispersal in tsetse (*Glossina* spp):**

**an artefact of inappropriate analysis**

**John W. Hargrove, John Van Sickle, Glyn A. Vale, Eric R. Lucas**

**S4 Text: Miscellaneous problems relating to the NDDD hypothesis**

**Index**

**Title Page**

A. Errors involved in the estimation of *b* 2

B. Errors involved in the estimation of *N_c_*, the true census density 6

C. Absence of correlation between $\hat{N}$*_c_* and $\hat{N}$*_e_* 10

D. Failure to allow for intensity and duration of trapping, differences in performance between traps, between species and between geographical/ecological regions 11

E. Contradictory evidence from trap catches 13

F. Inappropriate pooling of data for different situations 14

G. Unsupported claims of effects of NDDD reinvasion dynamics 15

H. No field evidence for larviposition pheromone in tsetse 16

I. Absence of any suggestion for a mechanism by which NDDD might have evolved 17

J. Errors in claimed support for NDDD 18

K. PDDD more likely than NDDD 18

L. Confusion between correlation and causation; possible reverse causality and confounding 19

**A. Errors involved in the estimation of *b***

Recall that *b* is the estimated slope of an isolation-by distance (IBD) regression, that is, a linear regression between genetic distance, *d_Gen_*, and the natural log of geographic distance, *d_geo_*. For each population, data on the genetic distance between flies sampled at two traps, and the corresponding geographic distance between them, is accumulated over multiple pairs of traps. A linear regression fitted to this data provides an estimate of *b* for that population.

The genetic distances in such data are not mutually independent because genetic information from any one trap may be included in multiple pairings with other traps. Thus, de Meeûs et al. (2019a) employed one of two nonstandard methods for testing the statistical significance of *b*. Where possible, they constructed an approximate 95% confidence interval (CI) for *b* using the bootstrap-over-loci (BOL) method (Leblois et al., 2003; de Meeûs et al. 2007). If the endpoints of the BOL CI were both positive, then they declared *b* to be statistically significant, which occurred in 7 of their 10 studies (Table A). For the remaining three studies, they instead conducted a Mantel randomization test of significance for the Pearson correlation between the two distance matrices (Legendre & Legendre, 1998; de Meeûs et al., 2007; Table A).

The choice, by de Meeûs et al. (2019a) to use either BOL or Mantel testing implies that these two methods are conceptually equivalent approaches for assessing the significance of *b*. However, this is not the case, because the BOL and Mantel methods address two different sources of uncertainty in *b*.

The first of these sources is sampling variation due to the small sample of loci from which all genetic distances are estimated for any one study. If a study had analysed a different sample of loci, it would yield different genetic distances, and hence a different estimate of *b*. This sampling variation can be modelled as measurement error in the response variable, *d_Gen_*, of the IBD regression. The BOL method addresses this source of variation by bootstrap resampling of the original sample of loci to generate replicate genetic distance matrices, which then provide replicate estimates of *b*.

However, the BOL method ignores the second source of uncertainty in *b*, which occurs because an IBD regression model does not fit its data perfectly, that is, *R^2^* < 1. Conventional significance testing and CIs for *b*, which are based on its standard error (SE), address this lack-of-fit uncertainty (Zar, 2010). These conventional methods are precluded, for IBD regressions, by non-independence of the genetic distances. However, the SE of *b* remains a useful quantifier of lack-of-fit uncertainty. The SE of *b* is directly proportional to the square root of the mean square (MSR) of the regression residuals, which are visualized as the scatter of points around the fitted regression. As exemplified by Fig A, IBD regressions often fit their data poorly: that is, *R^2^* is generally small and MSR is relatively large (Jenkins et al., 2010). For example, in 7 of the 10 de Meeûs et al. (2019a) studies for which we have data, the IBD regression has *R^2^* < 25%, and in 3 of those studies *R^2^* ≤ 1% (Table A). Thus, the lack-of-fit uncertainty in *b* will be substantial for most IBD regressions, unless the sample size is quite large.

The Mantel test accommodates non-independence in distance matrices, thus providing a valid test of significance for *b*, relative to its lack-of-fit uncertainty. However, the Mantel testing carried out by de Meeûs et al. (2019a) does not account for the sampled-loci source of uncertainty. In contrast, the BOL method treats the sampled-loci source of uncertainty in *b*, but does not account for the lack-of-fit source of uncertainty. It is clear that an accurate CI for *b* must account for both the sampled-loci and the lack-of-fit sources of uncertainty. Thus, either the BOL CI or the Mantel test, when performed by itself, is very likely to understate the true, overall uncertainty in *b*. As a result, one must seriously question the claims of statistical significance for all 10 of the *b* estimates from the 10 studies.

*Jackknife CIs for b*

We used jackknifing (Efron & Tibshirani, 1993; de Meeûs et al., 2007) to develop CIs of *b* for 7 of the de Meeûs et al. (2019a) studies for which we had data. Such CIs are an alternative to the Mantel test, for assessing the statistical significance of *b* relative to its lack-of-fit uncertainty. To deal with the non-independence of the genetic distances, we applied the jackknife’s “leave-one-out” approach to the individuals or subsamples for which multiple pairwise distances are calculated (Clarke et al., 2002). Thus, a single individual or subsample is removed from the regression data (“leave-one-out”) by omitting all genetic distances, and corresponding geographic distances, which involve that individual or subsample. For a data set based on pairwise distances among *n* individuals or subsamples, the jackknife estimator of *b*, and its approximate CI, are then derived using standard methods from *n* leave-one-out replicate regressions (Clarke et al., 2002, Efron & Tibshirani, 1993; Table A).

Three of our seven jackknifed CIs straddled zero (Table A), indicating non-significance of *b* relative to its lack-of-fit uncertainty. This result differs from the statistical significance implied by BOL CIs for these studies (Table A). For these three cases at least, the true CIs are likely to be somewhat wider than either their BOL CIs or their jackknifed CIs, and *b* would remain non-significant. If both sources of uncertainty could be accounted for, it is likely that some of the other *b*-values estimated by de Meeûs et al. (2019a) would also lose their statistical significance.


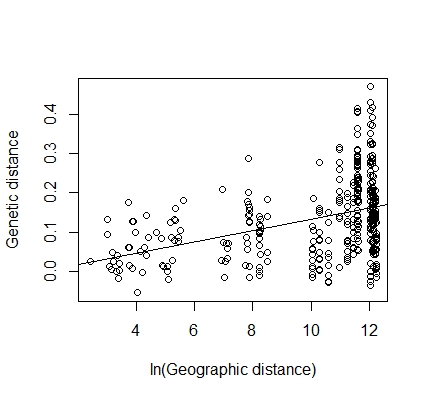


**Fig A** Data and fitted IBD regression for *G. tachinoides* study in Ghana. Row 2, Table A reports regression statistics (Adam et al., 2014).

**Table A. Significance testing of IBD regression slope, *b.*** de Meeûs et al. (2019a) supplied values of *b* and its CI, estimated via bootstrap-over-loci (BOL) for 7 studies, and also supplied Mantel test results for 3 studies. We calculated jackknife estimates of *b* and its 95% CI for 7 of the 10 studies, using genetic distances based on all available loci. We estimated squared Pearson correlations (*R^2^*) from all available genetic and geographic distances, with the full subset of loci included in genetic distances.

| Study | Species: Country | *b* point estimate  via BOL | 95% CI  via BOL | *b* point estimate via jackknife | 95% CI,  via jackknife | Number jackknife regressions | *R^2^* (percent) |
| --- | --- | --- | --- | --- | --- | --- | --- |
| Melachio et al. (2015) | *G. p. palpalis*: Cameroon | 0.0007 | 0.0006, 0.0014 | 0.0003 | -0.0071, 0.0076 | 16 | 0.1 |
| Adam et al. (2014) | *G. tachinoides*: Ghana | 0.0142 | 0.0076, 0.0229 | 0.0141 | 0.0057, 0.0225 | 27 | 15 |
| Hyseni et al. (2012) | *G. f. fuscipes*: Uganda | 0.0752 | 0.0325, 0.1301 | 0.0776 | 0.0298, 0.1253 | 19 | 48 |
| Manangwa et al. (2019) | *G. pallidipes*: Tanzania | 0.0168 | 0.0033, 0.0701 | -0.0022 | -0.0520, 0.0472 | 6 | 21 |
| Melachio et al. (2011) | *G. p. palpalis*: Cameroon | 0.0099 | 0.0060, 0.0170 | a | a | a | a |
| Okeyo et al. (2017) | *G. pallidipes*: Kenya/Nguruman | 0.0051 | 0.0019, 0.0113 | b | b | b | 20 |
| Manangwa et al. (2017) | *G. f. fuscipes*: Tanzania/Kenya | 0.0709 | 0.0286, 0.1290 | c | c | c | 72 |
| Koné et al. (2011) | *G. p. gambiensis*: Burkina Faso | 0.0146 | d | 0.0149 | -0.0081, 0.0379 | 31 | 1 |
| Koné et al. (2011) | *G. tachinoides*: Burkina Faso | 0.0146 | d | 0.0146 | 0.0002, 0.0290 | 37 | 0.9 |
| Opiro et al. (2017) | *G. f. fuscipes*: Uganda | 0.0202 | d | 0.0202 | 0.0061, 0.0344 | 33 | 22 |

a) Data not available

b) Sample size too small (6 distances)

c) Sample IDs missing from data, jackknifing not possible.

d) Mantel test, reported as significant by de Meeûs et al. (2019a)

**B. Errors in the estimation of *D_c_*, the true census density**De Meeûs et al. found a strong linear correlation (*R*^2^ = 0.86; *P*<0.02) between the logs of estimated dispersal distance ($\hat{}$) and census population density ($\hat{D}$*_c_* = $\hat{N}$*_c_*/$\hat{S}$), where the $\hat{N}$*_c_* values were derived from trap catches (De Meeûs et al., 2019a). As we shall now demonstrate, the errors in $\hat{D}$*_c_* are even more serious than those in $\hat{D}$*_e_*. We considered a model world consisting of a continuous population of tsetse covering a large area, say 100 km × 100 km = 10,000 km^2^. The population was uniform, across its entire extent, in terms of its true population density, which for the sake of illustration, we took as 1000 flies per km^2^. This assumption of fixed true density implies that the true effective density, *D_e_*, was also fixed, and hence δ was also fixed, assuming fixed *b*. Finally, we stipulated that a single trap, used in isolation, produces a daily catch of flies equal to 1% of the population in the surrounding 1 km^2^, i.e., 10 flies per trap per day in our model world.

 **Sampling the model population using more than one trap at a site** Using the de Meeûs et al., 2019a) definition for this case, *S* = π(*D_max_*)^2^, where *D_max_* is the distance between the two most distant traps in a given site. Note that this immediately assumes that the true area (*S*) of the subpopulation being sampled, and the surface area of the trapping site ($\hat{S}$), are identical. This assumption leads to problems. Suppose, for example, a subpopulation is sampled using six traps deployed as shown in Fig B(A), with a single trap placed at the centre a circle of radius *r*, and a further five traps placed at equally spaced distances along the circumference of the circle. This trap placement provides the most compact disposition conforming with the trap spacing used, for example, in Opiro et al. (2017).

Now consider the effect of changing the value of *r*, and thus *D_max_*, on the measured values of the census population ($\hat{N}$*_c_*) and the census population density ($\hat{D}$*_c_*) associated with the subpopulation being sampled. Initially, assume that there is a maximum distance of 10 m between traps, such that $\hat{S}$ (*r* ≈ 10 m) = π(*D_max_*)^2^ ≈ π(1.9 × 10)^2^ ≈1134 m^2^. Clearly, these closely packed traps will interfere with each other and the catch per unit time would probably differ little whether one used all six traps, or just the one at the centre – which would provide an expected catch of 10 flies per day if used in isolation anywhere in our population space (see above). The expected total catch per day from all six traps would thus be *N_c_* ≈ 10 flies. If *r* is increased to 30 m, such that $\hat{S}$ ≈ 10,000 m^2^ = 10^‑2^ km^2^, there will be less interference between the traps, but we expect that there will still be *some* interference and we thus expect $\hat{N}$*_c_* < 10 flies per trap per day, and the expected catch from all six traps will thus be 10 < $\hat{N}$*_c_* < 60 flies.

If *r* is increased to 100 m, as in Opiro et al. (2017), $\hat{S}$ (*r* = 100 m) ≈ 113,500 m^2^ = 0.114 km^2^, the traps are now sufficiently far apart that they are effectively acting independently [24]. Each trap is, therefore, expected to catch 10 flies per day, and the expected total daily catch will be $\hat{N}$*_c_* = 60 flies. For all further increases in trap spacing, and increases thereby in $\hat{S}$, there will be no further increase in $\hat{N}$*_c_* (Dransfield, 1984). Thus, the expected total daily catch is $\hat{N}$*_c_* = 60 flies – regardless of the value of *r*, as long as all traps used are separated from each other by distances of the order of at least 100 m. Since, however, the census population density is given by $\hat{D}$*_c_* = $\hat{N}$*_c_*/$\hat{S}$ it follows that, as *r* increases above 100 m, the measured value of $\hat{D}$*_c_* decreases as the square of the change in *r*. Thus, if we take $\hat{S}$(*r* =100 m) ≈ 0.114 km^2^ then $\hat{D}$*_c_* (*r* =100 m) = $\hat{N}$*_c_*(*r* =100 m)/$\hat{S}$(*r* =100 m) ≈ 60/0.114 ≈ 529 flies per km^2^. If, however, we double *r* such that $\hat{S}$(*r* = 200 m) ≈ 0.454 km^2^ then $\hat{D}$*_c_*(*r* =200 m) = $\hat{N}$*_c_*(*r* =200 m)/$\hat{S}$(*r* =200 m) = 60/0.454 ≈ 132 flies per km^2^, which is one quarter of the density for the situation where *r* = 100 m.

1.
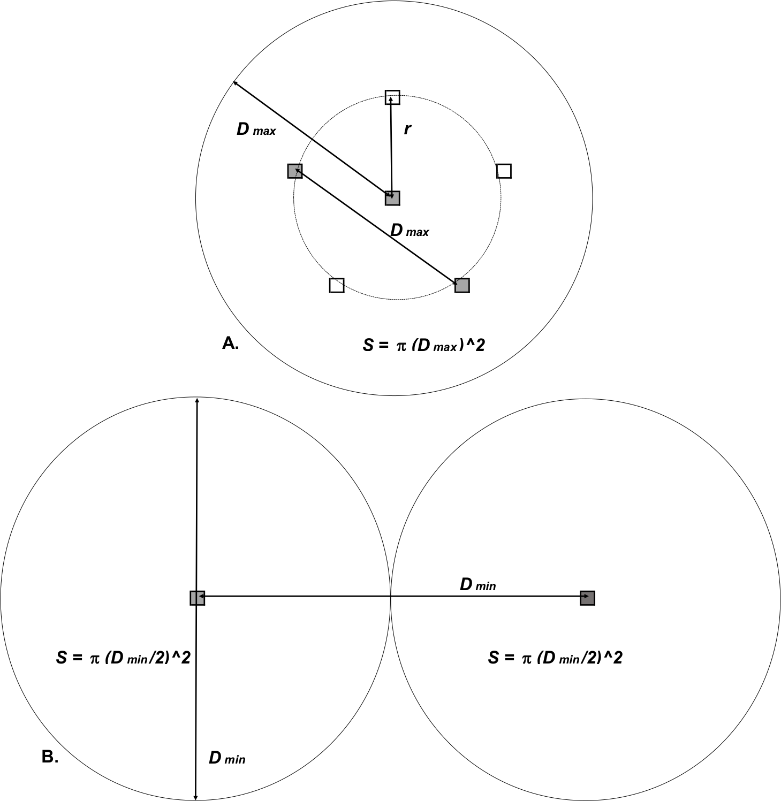


**Fig B. Surface area occupied by a subpopulation, as estimated using the NDDD protocol.** A. More than one trap deployed at a site. In the example shown (*cf* (Opiro et al., 2017) five traps are spaced at equal intervals on the circumference of a circle of radius *r* units, with a further trap at the centre of the circle. The protocol calculate the area of the site as $\hat{S}$ = π(*D_max_*)^2^, where *D_max_* is the distance between the two most distant traps in a given site, taken as the radius of the corresponding subpopulation. With this pattern, *D_max_* ≈ 1.9 *r*. B. Two trapping sites with one trap deployed at each site. For this scenario, the surface area occupied by the sub-population sampled is calculated from *S* = π(*D_min_*/2)^2^, where *D_min_* is the distance the distance between the centres of two neighbouring subpopulations and thus as the average diameter of a subpopulation.

**Sampling subpopulations using one trap at each site** Using the same model world as defined above, consider a situation where we sample two sites, using one trap at each site (Fig B(B)). The arguments follow the same course as for the situation where multiple traps are used at a single site. As before, the true value of δ is unaffected by the distance (*D_min_*) separating the centres of the neighbouring sub-populations. Moreover, the estimate of the census population number ($\hat{N}$*_c_*) is also independent of *D_min_*, and thus of $\hat{S}$. Since, however, *S* increases in proportion to the square of *D_min_*, it follows that the census population density (*D_c_* = *N_c_*/$\hat{S}$) decreases in proportion to the square of *D_min_*.

That is to say, for our model world, while the catch per trap per unit time is independent of *r* ≥ 100 m, the estimated population density declines with increasing *r* – whereas the true population density is constant, being independent of the pattern of trap deployment. At the same time, however, since the catch per trap is constant, the total catch increases in direct proportion to the number of traps deployed and the duration of deployment. As evidenced by the use made of data from Uganda (Opiro et al., 2017), de Meeûs et al. (2019a) took no account of these problems in their calculations of *N_c_* and *D_c_* for the various studies they used to derive the data for their Fig 1B. If this is the case, we must expect that the estimated values $\hat{N}$*_c_* and $\hat{D}$*_c_*, for any given study, bear little or no relation to the true values of population size and density for that study. Since, also, the various studies used to estimate $\hat{N}$*_c_* and $\hat{D}$*_c_* employed markedly different trap numbers and spacings, it follows that the ratio of these estimated values to the true values will be different in every study. These considerations cast considerable doubt on the validity of the values of $\hat{D}$*_c_* used by de Meeûs et al. (2019a) on the abscissa in their Fig 1B.

The implications of the above problems with $\hat{D}$*_c_* are again exemplified by contrasting the results from Uganda and Tanzania (Opiro et al., 2017; Manangwa et al, 2019). For the former, de Meeûs et al. use $\hat{N}$*_c_* = 108, in accord with the fact that six traps, placed at least 100 m from each other, caught an average of 18 flies per trap over a period of 3-4 days, or about 5 flies per trap per day (de Meeûs et al., 2019a; Opiro et al, 2017). Using the de Meeûs et al. (2019a) estimate of $\hat{S}$ = 0.02 km^2^ for this study, $\hat{D}$*_c_* = $\hat{N}$*_c_*/$\hat{S}$ = 108/0.02 = 5400 tsetse per km^2^. De Meeûs et al. do not quote a value of $\hat{D}$*_c_* for the Tanzania study (Manangwa et al., 2019) – but we know that two traps were used at each site and that each trap caught about 20 flies per day. For comparison with the Uganda study Opiro et al. (2017), suppose the total catch from the two traps over a 3-day period was then 2×3×20 = 120 flies. Using the de Meeûs et al. (2019a) estimate of $\hat{S}$ ≈ 34.87 km^2^ for this study, gives $\hat{D}$*_c_* = $\hat{N}$*_c_*/$\hat{S}$ = 120/34.87 = 3.44 flies per km^2^. We thus have the anomalous situation where the calculated census density is 5400/3.44 ≈ 1569 times higher in Uganda than in Tanzania, despite the catch in Uganda being a quarter, i.e., 5/20, of that in Tanzania. The apparent implication is that the availability to traps varies by at least 6000-fold between the two study areas, thereby suggesting that trap catches are hopeless indices of tsetse abundance. That alone is sufficient to render Fig 1B of de Meeûs et al. (2019a) entirely meaningless, although few field entomologists would credit that trap performances differ quite so markedly. The main problem, which is equally damaging, is probably erroneous estimates of $\hat{D}$*_c_* stemming from gross errors in $\hat{S}$.

**C. Absence of correlation between** $\hat{\boldsymbol{N}}$***_c_* and** $\hat{\boldsymbol{N}}$***_e_***

De Meeûs et al. (2019a) state that they expect a strong correlation between the true values of *N_e_* and *N_c_*. Otherwise – as they say – all population genetics studies of tsetse would need to be called into question. Their data do not, however, support their expectation. The linear correlation between their estimates of $\hat{N}$*_c_* and $\hat{N}$*_e_* for the tsetse populations used in their own study is only 0.39 and is not significantly different from zero (Fig C(A)). Moreover, whereas the authors claim that *N_e_* values should be less than *N_c_*, in reality *N_c_* < *N_e_* in five out of six situations where they provide data for the two variables (Table A of S1 Table). Notice, however, that if we divide each $\hat{N}$*_c_* and $\hat{N}$*_e_* value by $\hat{S}$, to create the population densities, $\hat{D}$*_c_* and $\hat{D}$*_e_*, there is a very high correlation between the log transformed versions of these two variables (Fig C(B)). This is consistent with our earlier suggestion that many of the results are artefacts resulting from inappropriate estimates of *S*, which varies over a range large enough to swamp all other sources of variation. Nonetheless, the following section suggests that there are additional good reasons to expect serious errors in the estimates of *N_c_*.


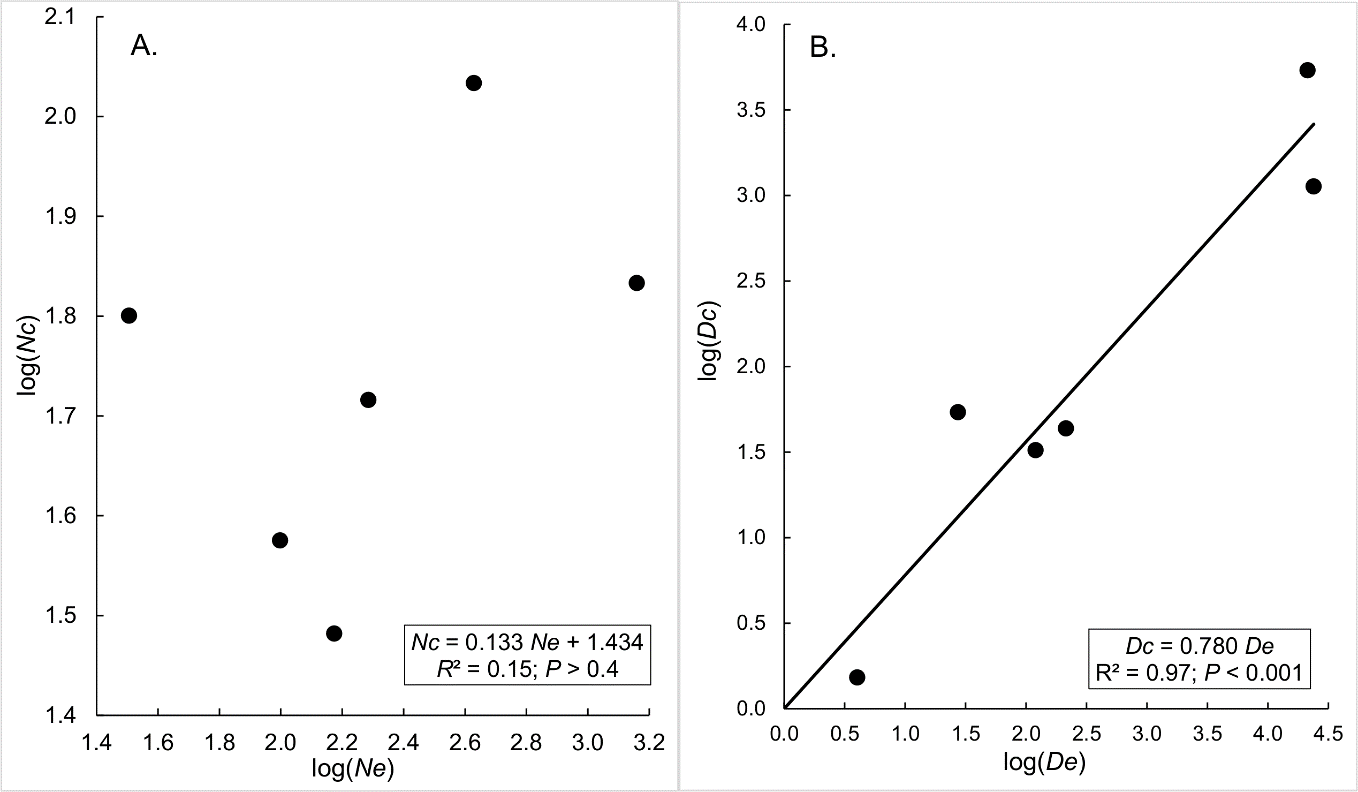


**Fig C.** **Effective and census population numbers and densities using data from de Meeûs et al. (2019a).** Plots of: A. census population size ($\hat{N}$*_c_*) against effective population size ($\hat{N}$*_e_*); B. census population density ($\hat{D}$*_c_*) against effective population density ($\hat{D}$*_e_*). All data transformed by taking logs to the base 10.

**D. Failure to allow for intensity and duration of trapping, differences in performance between traps, between species and between geographical/ecological regions** Scrutiny of Opiro et al. (2017) exposes further problems with the de Meeûs et al. (2019a) estimates of census numbers, $\hat{N}$*_c_* and density $\hat{D}$*_c_*. As detailed above, the value of $\hat{N}$*_c_* = 108 for this study reflects the number of flies captured per site – regardless of the number of traps used, and without regard to the trapping period. Failure to adjust for trapping intensity in different studies presents a serious problem. For example, with reference to Fig B(A), suppose the three traps at the white-filled squares were removed. Clearly, the expected catch – and thus $\hat{N}$*_c_* – would be halved. For the three traps remaining at the grey-filled square, however, *D_max_* – and thus $\hat{S}$ – would be unchanged, so that$\hat{D}$*_c_* would be halved.

Moreover, there appears to have been no attempt to adjust for the trapping duration, which is approximated as an ill-defined 3-4 days. If the traps had only been run for one day, the expected catch, and $\hat{N}$*_c_*, would have been reduced by around two-thirds or three-quarters. The de Meeûs et al. (2019a) paper and its Supplementary Materials provide no suggestion, for any of the study sites considered, that the catches were standardised relative to the number of traps employed and the duration of trapping.

De Meeûs et al. (2019a) cite Barclay & Hargrove (2005) as support for their claim that the census of flies captured in the studies they used were correlated with the real census of the corresponding tsetse populations. This is inappropriate, for two reasons. First, the paper cited provides no tsetse census data – real or otherwise. Second, it presents data only for *G. m. morsitans* and *G. pallidipes*; de Meeûs et al. (2019a) do not provide census population estimates for either species, and neither species is represented in their Fig 1B. For both of these reasons their census results cannot justifiably be correlated with tsetse census data from the paper cited. What Barclay & Hargrove (2005) did suggest, was that there was an order of magnitude difference between the probability of capturing *G. m. morsitans* and *G. pallidipes* in an odour-baited trap. This underlines the danger of assuming, as de Meeûs et al. (2019a) have done implicitly in their Fig. 1B, that differences in trap catches of different species are correlated with differences between the true population densities for those species.

In offering these estimates of capture probability, Barclay & Hargrove (2005) were careful to stipulate that all catches referred to a single device, run for a one-day sampling period. They also expressed the probabilities as a percentage of the population of flies occurring in a 1-km^2^ neighbourhood of the sampling device. As detailed above, de Meeûs et al. (2019a) failed to correct the trap catches they used for trap type, trapping duration, numbers of traps, or the number of flies in a well-defined neighbourhood of the trap.

Even if they had corrected the catch for the intensity and duration of trapping , there is still the problem that the studies considered by de Meeûs et al. (2019a) employed a variety of trapping techniques, involving different sorts of trap used to catch different species of tsetse. This is important because the design of traps and the species against which they are deployed can affect, by at least one order of magnitude, the numbers caught (Challier & Laveissière, 1973; Hargrove, 1977, 1980; Mihok, 2002). The problem is aggravated further by the fact the some of the traps in the studies analysed by de Meeûs et al. (2019a) were employed with highly effective odour attractants (Manangwa et al., 2019), and others were not (Melachio et al., 2015). In view of all of the above problems, it appears that the trap catches, as employed by de Meeûs et al. (2019a), are meaningless indices of population numbers and density.

**E. Contradictory evidence from trap catches** The de Meeûs et al. (2019a) results often contradict common sense. An extreme example is provided by the estimates derived using the Opiro et al. (2017) study on *G. fuscipes fuscipes* in Uganda. The very low value of $\hat{S}$ = 0.02 km^2^, coupled with almost the highest value of $\hat{D}$*_e_* >21,000 tsetse per km^2^ among all 10 studies, led to an absurdly low estimate of $\hat{}$ = 27 m for the dispersal per generation. With a 60-day generation time assumed by de Meeûs et al. (2019a), this is equivalent to a daily flight distance of about 4 m. This makes no sense: female tsetse must locate and feed on a host at least three times in a 9-day inter-larval period if they are to produce a healthy pupa (Langley & Stafford, 1990; Randolph et al., 1992). This seems virtually impossible if a fly moves only 4 m each day. The very low rate of movement is also in stark contrast to a mark-recapture estimate of 338 m/day for *G. f*. *fuscipes* in Uganda (Rogers, 1977), >80 times higher than the de Meeûs et al. (2019a) estimate, and equivalent to a dispersal of δ = 1.9 to 2.6 km per generation.

Moreover, if de Meeûs et al. (2019a) are correct in claiming that census population density is at least as large as effective population density, then we might also expect that the true absolute density of tsetse in the area studied by Opiro et al. (2017) could be of the order of 21,000 per km^2^. However, this makes little sense given that the results in Opiro et al. (2017) show that tsetse catches averaged only 5 per trap per day. By contrast, in Rogers’ (1977) a single team of stationary men caught tsetse at the rate of 120 *per* *hour*, suggesting that the actual population density could have been 2-3 orders of magnitude higher than for the Opiro et al. (2017) study area. The problem then is that if, as suggested by the NDDD hypothesis, dispersal rates decrease markedly with increasing population density, expected dispersal rates should have been far lower in the study of Rogers (1977) than in Opiro et al. (2017) study, rather than the reverse.

Similar concerns are raised by the estimates derived from the study on *G. pallidipes* (Manangwa et al., 2019). Here the effective population density is quoted as $\hat{D}$*_e_* = 1.3 tsetse per km^2^, four orders of magnitude lower than the value estimated by de Meeûs et al. (2019a) from the study on *G. f*. *fuscipes* (Opiro et al., 2017). It then makes no sense that tsetse catches were about four times higher in the Manangwa et al. (2019) study than in Opiro et al. (2017). These matters all make sense, however, when one realizes that the $\hat{D}$*_e_* and $\hat{D}$*_c_* estimates in the de Meeûs et al. (2019a) analysis are essentially artefacts of the gross variation in trap placements and the huge resultant errors in $\hat{S}$.

**F. Inappropriate pooling of data for different situations** The de Meeûs et al. (2019a) study involved tsetse populations in ten locations, in six countries. We question the validity of performing a pooled analysis on such an *ad hoc* collection of results that refer to a variety of tsetse species in different circumstances. The pertinent question is, rather, how dispersal rates might change with the variation in population density for a given species in a given place from time to time. Hence, when de Meeûs et al. (2019a) interpret their Fig 1 as they do, they must have made the assumption that the relationship between density and dispersal rate applies to all species in all circumstances at all times. Indeed, somewhat strangely, after making this assumption to interpret their results, they then conclude that their interpretations support the assumption. However, that assumption is invalidated by our consideration (see above) of the way that the estimates of *D_e_* and *D_c_* vary due to sampling procedures and species behaviour at any given place and time. Furthermore, there are many variables other than population density that are likely to affect mobility substantially, including vegetation type and fly size (Vale et al., 2014), and perhaps also climate, and the type and abundance of hosts and sympatric species of tsetse and other biting flies.

Even if we did give credence to the procedures used by de Meeûs et al. (2019a), we would have to accept that their Fig 1 would make sense only if the habitat and population density were each homogeneous within each of the ten situations considered. Most situations in tsetse belts are not like that. For example, in the study area for *G. f. fuscipes* in Northern Uganda the terrain is stated to be very heterogeneous (Opiro et al., 2017). In such conditions it is usual to place traps where experience has suggested that catches will be the greatest, ignoring the much larger areas where catches per trap are lower, but where a substantial part of the population is likely to be present at low density. Hence, to what part of the study area, if any, do the outputs for dispersal rate and density refer?

**G. Unsupported claims of effects of NDDD reinvasion dynamics** When discussing possible causes of NDDD and asserting that dispersal is strongly density-dependent in tsetse, de Meeûs et al. (2019a) pay no attention to the dynamics of altered dispersal that are predicted by their Equation (1). Similarly, in their abstract and on p.5, they assert that “… control campaigns might unleash dispersal from untreated areas.” This could occur only if tsetse were reacting to the population density in the destination of their movement, i.e., the treated area, rather than in their current, untreated, location. However, this model of movement contravenes the assumptions of the underlying model (Rousset, 1997), which clearly requires that the dispersal and density parameters must describe the same population. Thus, given that an untreated area would have a much higher population density than a treated area, flies in the former would have a lower dispersal rate than flies in the treated area. Control would not, therefore, be expected to “unleash dispersal”, that is, rapid reinvasion. De Meeûs et al. (2019a) also suggest that “… the bigger the decrease in the (treated) population, the higher potential for reinvasion…”, and that this is also due to density dependence. However, this dynamic, even if correct, is not implied by their model (Equation (1)). That model says nothing about a population’s potential for reinvasion.

**H. No field evidence for larviposition pheromone in tsetse** In apparent justification of the idea that females might return to previous larviposition sites, de Meeûs et al. (2019a) claim that *G. morsitans* in the field secrete a larviposition pheromone that attracts other females to the same site, leading to a strong aggregation of pupae (WHO, 2013). Inspection of the work cited shows, however, that it makes no mention of pheromones of any sort. Laboratory evidence for such a pheromone has indeed been published (Saini et al., 1996; Renda et al., 2016; Gimonneau et al., 2020) but no such chemical has ever been shown to produce aggregation of pupae in the field. Nonetheless, the suggestion by de Meeûs et al. (2019a) that tsetse dispersal is in some way linked to the existence of a larval deposition pheromone has been reiterated by some of the co-authors (Gimonneau et al., 2020). The arguments adduced in support of this idea are, however, confused or unconvincing in several ways. First, allowing that the predation of tsetse pupae increases with the local density of the pupae in the wild (Rogers & Randolph, 1984), deliberate aggregation of pupae would be expected to reduce, rather than increase, survival probability. Second, there is the potential problem that predators could benefit from a signal that pupae occur at a particular site – pupae are best hidden, not advertised. Finally, and most confusingly, it is claimed that the pheromone would have its greatest benefit at low population densities, because sparse populations become extinct if they disperse rapidly (Gimonneau et al., 2020). This contradicts completely the de Meeûs et al. (2019a) idea that sparse populations evolve to disperse widely: in short, the co-authors are themselves arguing against their own notion of NDDD.

**I. Absence of any suggestion for a mechanism by which NDDD might have evolved** Whether or not we allow that the population density within each study area is varied or uniform, there is a problem in seeing a credible biological mechanism for the occurrence of the NDDD apparent in Fig 1 of de Meeûs et al. (2019a). When asked for their understanding of this, all that was offered in response was that evolution was involved, with no explanation of how such evolution was driven (Lord, 2019; de Meeûs et al., 2019b). However, the idea of a particular dispersal rate evolving in response to a specific density in a given situation, as conceived in de Meeûs et al. (2019a) Fig 1, creates a dilemma. On one hand, the plots of density vs dispersal in their Fig 1 would be nonsensical if the density were variable at any one time within the study area covered by each plot. On the other hand, if density were uniform within each study area at a given time, then density could not exert any direct selection pressure, since however much the flies moved, they would experience the same population density – they would not find densities more favourable to survival.

As an alternative, a combination of three phenomena might be proposed: (i) that tsetse have some optimal density, (ii) natural selection has resulted in tsetse behaving in such a way as to tend to stabilise the density at that optimum value, and (iii) the optimal density is achieved via changes in the dispersal rate, in direct response to the population density experienced by the flies. Any such change in dispersal rate would have to operate by increasing dispersal when local densities are above the optimum, and causing aggregation when local densities are sub-optimal. However, the dispersal mechanism just described is clearly PDDD, not NDDD. It is thus most confusing that, as indicated above, at least some of the co-authors on the NDDD paper appear eager to promote this idea (Gimonneau et al., 2020).

**J. Errors in claimed support for NDDD** De Meeûs et al. (2019a) cite various works in arguing that NDDD in tsetse populations has been known for some time (WHO, 2013; Rogers & Randolph, 1984; Vale, 1974; Randolph et al., 1984; Bouyer et al., 2007). None of the papers cited, however, make any explicit or even implicit mention of NDDD; nor do they provide any support for the idea. As an example, Rogers & Randolph (1984) are cited in support of the principle that the efficacy of population suppression may be reduced at low densities if normal density-dependent constraints are removed. This idea has long been accepted by the generality of tsetse workers, but it does not imply any recognition of NDDD.

**K. PDDD more likely than NDDD** There is no credible evidence from field work with tsetse that control operations, which often cause huge reductions in population densities, result in increased dispersal rates through NDDD. In a critique of NDDD in tsetse, Lord (2019) advanced an evidence-based argument that control operations might actually have the very opposite effect, causing what is effectively PDDD. The reasoning was that tsetse control campaigns, which raise the death rate among adult tsetse, are associated with a marked increase in the proportion of young adult females in the tsetse population (Van Sickle & Phelps, 1988), and such flies are unlikely to disperse far since their flight capacity is poor (Hargrove, 1975). This argument is certainly no evidence that a reduction in density will itself cause a decreased dispersal, but it does weaken further the claim that control measures will cause problems by enhancing dispersal. De Meeûs et al. (2019b) made no effort to refute the argument by Lord (2019).

**L. Confusion between correlation and causation; possible reverse causality and confounding** Even if we disregard all of the many problems detailed above, and allow that the correlations apparent in Fig. 1 of de Meeûs et al. (2019a) are valid, correlation does not necessarily imply causation. Note that the gene flow theory behind Equation (1) treats dispersal and density as independent population parameters, with no suggestion of a mechanistic relationship between them (Rousset, 1997). Moreover, even if we accepted the idea of a dependent relationship between these parameters, we could not be sure whether that meant that declining density caused increasing dispersal, or that increased dispersal caused decreased density, or that the levels of density and dispersal were each caused independently by one or more other factors. Given all of these doubts about any causal background to their Fig. 1, we conclude that de Meeûs et al. (2019a) have failed to produce evidence that tsetse control in any one place will induce *any* increase in the dispersal rate – let alone a *gross* increase. It is also presumptuous to predict that the claimed causal relationship will apply to all species of tsetse everywhere.

**References**

**Adam, Y., Bouyer, J., Dayo, G.K., Mahama, C.I., Vreysen, M.J., Cecchi, G., Abd-Alla, A.M., Solano, P., Ravel, S., de Meeûs, T.** (2014) Genetic comparison of *Glossina tachinoides* populations in three river basins of the upper west region of Ghana and implications for tsetse control. *Infection, Genetics and Evolution*, **28**, pp.588-595.

**Barclay, H.J. & Hargrove, J.W.** (2005) Probability models to facilitate a declaration of pest-free status, with special reference to tsetse (Diptera: Glossinidae). *Bulletin of Entomological Research* **95**, 1-9.

**Bouyer, J., Pruvot, M., Bengaly, Z., Guerin, P. M., & Lancelot, R.** (2007). Learning influences host choice in tsetse. *Biology Letters*, **3**, 113-117.

**Challier, A. & Laveissière, C.** (1973) Un nouveau piège pour la capture des glossines (*Glossina*: *Diptera, Muscidae*) déscription et essais sur la terrain. *Cahiers de l'ORSTOM, Série Entomologie Médicale et Parasitol*ogie **11**, 251-262.

**Clarke, R.T., Rothery, P., Raybould, A.F.** (2002) Confidence limits for regression relationships between distance matrices: Estimating gene flow with distance. *Journal of Agricultural, Biological and Environmental Statistics* **7**, 361-372.

**De Meeûs, T., McCoy, K.D., Prognolle, F. Chevillon, C., Durand, P., Hurtrez-Boussès, S., Renaud, F.** (2007) Population genetics and molecular epidemiology, or how to “débusquer la bête”. *Infection, Genetics and Evolution* **7**, 308-332.

**De Meeûs T., Ravel S., Philippe Solano P., Bouyer J.** (2019a) Negative density-dependent dispersal in tsetse flies: a risk for control campaigns? *Trends in Parasitology* **35**, 615-621. [**https://doi.org/10.1016/j.pt.2019.05.007**](https://doi.org/10.1016/j.pt.2019.05.007)

**De Meeûs, T., Ravel, S., Solano, P., & Bouyer, J. (**2019b) Response to the Comments of J.S. Lord. *Trends in Parasitology*, **35**, 742.

**Dransfield, R. D.** (1984). The range of attraction of the biconical trap for *Glossina pallidipes* and *Glossina brevipalpis*. *International Journal of Tropical Insect Science*, **5**, 363-368.

**Efron, B., Tibshirani, R.J.** (1993) *An Introduction to the Bootstrap*. Chapman-Hall, London.

**Gimonneau, G., Ouedraogo, R., Salou, E., Rayaisse, J. B., Buatois, B., Solano, P., Dormont, L., Roux, O. & Bouyer, J.** (2020). Larviposition site selection mediated by volatile semiochemicals of larval origin in *Glossina palpalis gambiensis*. *Ecological Entomology*, [**https://doi.org/10.1111/een.12962**](https://doi.org/10.1111/een.12962)

**Hargrove**, **J. W.** (1975) The flight performance of tsetse flies. *Journal of Insect Physiology* **21**, 1385-1395.

**Hargrove**, **J. W.** (1977) Some advances in the trapping of tsetse (*Glossina* spp.) and other flies. *Ecological Entomology*, **2**, 123-137.

**Hargrove, J. W.** (1980) Improved estimates of the efficiency of traps for *Glossina morsitans morsitans* Westwood and *G. pallidipes* Austen (Diptera, Glossinidae), with a note on the effect of the concentration of accompanying host odour on efficiency. *Bulletin of Entomological Research*, **70**, 579-587.

**Hyseni, C., Kato, A.B., Okedi, L.M., Masembe, C., Ouma, J.O., Aksoy, S. and Caccone, A.** (2012) The population structure of G. fuscipes fuscipes in the Lake Victoria basin in Uganda: implications for vector control. *Parasites and Vectors*, **5**:222, 1-14. [**http://www.parasitesandvectors.com/content/5/1/222**](http://www.parasitesandvectors.com/content/5/1/222)

**Jenkins, D.G., Carey, M., Czerniewska, J., Fletcher, J., Hether, T., Jones, A., Knight, S., Knox, J., Long, T., Mannino, M., McGuire, M., Riffle, A., Segelsky, S., Shappell, L., Sterner, A., Strickler, T., Tursi, R.** (2010) A meta-analysis of isolation by distance: relic or reference standard for landscape genetics? *Ecography* **33**, 315-320.

**Koné, N., Bouyer, J., Ravel, S., Vreysen, M.J., Domagni, K.T., Causse, S., Solano, P., De Meeûs, T. (**2011) Contrasting population structures of two vectors of African trypanosomoses in Burkina Faso: consequences for control. *PLoS Neglected Tropical Diseases*, **5**(6) e1217. [**https://doi:10.1371/journal.pntd.0001217**](https://doi:10.1371/journal.pntd.0001217)**.**

**Langley, P. A. & Stafford, K.** (1990) Feeding frequency in relation to reproduction in *Glossina morsitans morsitans* Westwood and *G. pallidipes*. *Physiological Entomology*, **15**, 415-421.

**Leblois, R., Estoup, A., Rousset, F.** (2003) Influence of mutational and sampling factors on the estimation of demographic parameters in a “continuous” population under isolation by distance. *Molecular Biology and Evolution* **20**, 491-502.

**Legendre, P. Legendre, L.** (2012) *Numerical Ecology* *(3^rd^ ed.)* Elsevier, Amsterdam.

**Lord, J. S.** (2019). Comments on T. De Meeûs et al.’s Article. *Trends in Parasitology*, **35**, 741-742.

**Manangwa, O., Nkwengulila, G., Ouma, J.O., Mramba, F., Malele, I., Dion, K., Sistrom, M., Khan, F., Aksoy, S. and Caccone, A.** (2017) Genetic diversity of *Glossina fuscipes* *fuscipes* along the shores of Lake Victoria in Tanzania and Kenya: implications for management. *Parasites and Vectors*, **10**:268, 1-8.

**Manangwa, O., De Meeûs, T., Grébaut, P., Ségard, A., Byamungu, M., Ravel, S.** (2019). Detecting Wahlund effects together with amplification problems: cryptic species, null alleles and short allele dominance in *Glossina pallidipes* populations from Tanzania. *Molecular Ecology Resources*, **19**, 757-772.

**Melachio, T. T., Simo, G., Ravel, S., De Meeûs, T., Causse, S., Solano, P., Lutumba, P., Asonganyi, T., Njiokou, F.** (2011). Population genetics of *Glossina palpalis palpalis* from central African sleeping sickness foci. *Parasites & Vectors***, 4**, 140.

**Melachio, T. T., Njiokou, F., Ravel, S., Simo, G., Solano, P., De Meeûs, T.** (2015) Effect of sampling methods, effective population size and migration rate estimation in *Glossina palpalis palpalis* from Cameroon. *Infection, Genetics and Evolution*, **33**, 150-157.

**Mihok, S.** (2002). The development of a multipurpose trap (the Nzi) for tsetse and other biting flies. Bulletin of entomological research, **92**, 385-403.

**Okeyo, W.A., Saarman, N.P., Mengual, M., Dion, K., Bateta, R., Mireji, P.O., Okoth, S., Ouma, J.O., Ouma, C., Ochieng, J., Murilla, G. (**2017) Temporal genetic differentiation in *Glossina pallidipes* tsetse fly populations in Kenya. *Parasites & Vectors*, **10**:471, 1-13. [**https://doi.org/10.1186/s13071-017-2415-y**](https://doi.org/10.1186/s13071-017-2415-y)

**Opiro, R., Saarman, N.P., Echodu, R., Opiyo, E.A., Dion, K., Halyard, A., Dunn, A.W., Aksoy, S., Caccone A.** (2017) Genetic diversity and population structure of the tsetse fly *Glossina fuscipes fuscipes* (Diptera: Glossinidae) in Northern Uganda: Implications for vector control. *PLoS Neglected Tropical Diseases* **11(4)**: e0005485. [**https://doi.org/10.1371/journal.pntd.0005485n**](https://doi.org/10.1371/journal.pntd.0005485n)

**Randolph, S. E., Rogers, D. J. & Kuzoe, F. A. S.** (1984) Local variation in the population dynamics of *Glossina palpalis palpalis* (Robineau-Desvoidy) (Diptera: Glossinidae). II. The effect of insecticidal spray programmes. *Bulletin of Entomological Research*, **74**, 425-438.

**Randolph, S.E., Williams, B.G., Rogers, D.J. & Connor, H.** (1992) Modelling the effect of feeding-related mortality on the feeding strategy of tsetse (Diptera: Glossinidae). *Medical and Veterinary Entomology* **6**, 231-240.

**Renda, S., De Beer, C. J., Venter, G. J., & Thekisoe, O. M. M.** (2016). Evaluation of larviposition site selection of *Glossina brevipalpis*. *Veterinary Parasitology*, **215**, 92-95.

**Rogers, D.** (1977) Study of a natural population of *Glossina fuscipes fuscipes* Newstead and a model of fly movement. *Journal of Animal Ecology*, **46**, 309-330.

**Rogers, D. J. & Randolph, S. E.** (1984) A review of density-dependent processes in tsetse populations. *Insect Science and its Application*, **5**, 397-402.

**Rousset, F.** (1997) Genetic differentiation and estimation of gene flow from F-statistics under isolation by distance. *Genetics* **145**, 1219-1228.

**Saini, R.K., Hassanali, A., Andoke, J., Ahuya, P. & Ouma, W.P.** (1996) Identification of major components of larviposition pheromone from larvae of tsetse flies *Glossina morsitans morsitans* Westwood *Glossina morsitans centralis* Machado. *Journal of Chemical Ecology*, **22**, 1211-1220.

**Vale, G. A.** (1974) Direct observations on the responses of tsetse flies (Diptera: Glossinidae) to hosts. *Bulletin of Entomological Research*, **64**, 589-594.

**Vale G.A., Hargrove J.W., Solano P., Courtin F., Rayaisse J-B., Lehane M.J., Esterhuizen J., Tirados N., Torr S.J.** (2014) Explaining the host-finding behavior of blood-sucking insects: computerized simulation of the effects of habitat geometry on tsetse fly movement. *PLoS Neglected Tropical Diseases* 8(6): [**https://doi:10.1371/journal.pntd.0002901**](https://doi:10.1371/journal.pntd.0002901).

**Van Sickle, J. & Phelps, R. J.** (1988) Age distributions and reproductive status of declining and stationary populations of *Glossina pallidipes* Austen (Diptera: Glossinidae) in Zimbabwe. *Bulletin of Entomological Research*, **78**, 51-61.

**WHO** (2013) Control and Surveillance of Human African Trypanosomiasis*: Report of a WHO Expert Committee, World Health Organization & WHO Expert Committee on the Control and Surveillance of Human African Trypanosomiasis, WHO.* [**http://www.who.int/iris/handle/10665/95732**](http://www.who.int/iris/handle/10665/95732)

**Zar, J.H.** (2010) *Biostatistical Analysis (5^th^ ed.)* Prentice Hall, Upper Saddle River, NJ, USA.
