# Supplementary material for: Negative density-dependent dispersal in tsetse (Glossina spp): An artefact of inappropriate analysis
Source: PLoS Negl Trop Dis. 2021 Mar 25;15(3):e0009026. doi: 10.1371/journal.pntd.0009026 (PMC8023489; doi:10.1371/journal.pntd.0009026)
Supplement: S2 Text — (DOCX) [file pntd.0009026.s005.docx]

**Negative density-dependent dispersal in tsetse (*Glossina* spp);**

**an artefact of inappropriate analysis**

**J. W. Hargrove, J. Van Sickle, G. A. Vale, E. R. Lucas**

**S2 Text: Mathematics of the “errors-in-D_e_” argument**

Equation 1 can be written in log-transformed format as:

*log(δ) = C – 0.5*log(D_e_) - 0.5*log(b)* (S2-1).

In Equation S2-1, *C* is a constant. The parameters *δ, D_e_*, and *b* are assumed to be “true” values, that is, having zero estimation error.

De Meeûs et al. (2019a) do not know the true values of these parameters for their 10 populations. Instead, insert estimated values *D̂_e_* and *b̂* into Equation S2-1:

*log(δ̂) = C – 0.5*log(D̂_e_) - 0.5*log(b̂)* (S2-2).

Thus, estimation errors in *D̂_e_* and *b̂* are propagated into the prediction of dispersal, resulting in an estimated value *δ̂* which also may be erroneous. We assume multiplicative error models for *D_e_* and *b.* That is, assume that the estimated values of these parameters are:

*D̂_e_ = D_e_*ε_D_* and *b̂ = b*ε_b_* , where the errors *ε_D_* and *ε_b_* are random.

Substitution for *D̂_e_* into Equation S2-2 yields:

*log(δ̂)* = *C - 0.5*log(D_e_) - 0.5*log(ε_D_*) *- 0.5*log(b̂)* (S2-3).

And this can be further expanded to yield:

*log(δ̂)* = *C - 0.5*log(D_e_) - 0.5*log(ε_D_*) *- 0.5*log(b)* *- 0.5*log(ε_b_*). (S2-4).

We now show how Equation S2-4 generalizes our simulations, in which *D_e_* was assumed to be estimated with random error.

In those simulations, we first assumed a fixed value *δ*, while *D_e_* was allowed to vary, across populations. Thus, *log(δ) = A* where *A* is a constant, across a set of populations. This assumes there is not a density-dependent relationship between *δ* and *D_e._*  Given any set of true *D_e_* values, the true *b* parameter is assumed to adjust, via Equation S2-1, to maintain constancy in *δ*. With these assumptions, Equation 1 becomes:

*A = C – 0.5*log(D_e_) - 0.5*log(b)* (S2-5).

Now, substitute *A* for three of the terms in Equation S2-4. The result is:

*log(δ̂)* = *A* - *0.5*log(ε_D_*) *- 0.5*log(ε_b_*) (S2-6).

Equation S2-6 shows that, under an assumption of fixed *δ* and no density dependence, the variation in error-contaminated predictions of *δ,* across populations, is driven entirely by the errors in *D_e_* and *b*. For simplicity, the simulations also assume that estimation of *b* is error-free, that is, *b̂* *= b*. Under this assumption, *ε_b_* *= 1* for all populations, and Equation S2-6 simplifies to:

*log(δ̂)* = *A* - *0.5*log(ε_D_*) (S2-7).

Our simulations also assume that the estimation errors *ε_D_* have much greater variability across populations than does *D_e_* itself. With this assumption, the product *D_e_*ε_D_* can be approximated by *H***ε_D_*, where *H* is a constant. Thus, *log(ε_D_*) ≈ *log(D̂_e_) - log(H).* Assuming this approximation, Equation S2-7 becomes:

*log(δ̂)* ≈ (sum of constants) - *0.5*log(D̂_e_)* (S2-8).

In other words, *log(δ̂)* will be approximately linearly related to *log(D̂_e_)*, with a slope of -0.5. This result is the main outcome of our simulations.

Our error model also explains why one sees a strong, positive correlation between *log(δ̂)* and *log(S)* in the data from de Meeûs et al. (2019a). Assume that *log(ε_D_*) has much greater variation, across the 10 populations, than do *log(D_e_)* and *log(b̂).* If this is true, then *log(D_e_)* and *log(b̂)* behave almost like constants, relative to the variation in *log(ε_D_*) across the 10 populations. Thus, Equation S2-3 can be approximated by

*log(δ̂)* ≈ *(sum of constants) - 0.5*log(ε_D_*) (S2-9).

That is, we would expect to see an approximate linear relationship between *log(δ̂)* and *log(ε_D_*), with a slope of -0.5.

Next, also assume that the errors *ε_D_* are due almost entirely to *S*. If so, then *ε_D_* ≈ *k/S*, where *k* is a constant. Substitution into Equation S2-9 yields:

*log(δ̂)* ≈ *(sum of constants) + 0.5*log(S*) (S2-10).

Indeed, Figure 1B shows exactly this relationship, supporting our contention that the estimation errors *ε_D_* have much greater variation than *D_e_*, and that those errors are due primarily to the values of *S*.

**Reference**

**De Meeûs T., Ravel S., Philippe Solano P., Bouyer J.** (2019a) Negative density-dependent dispersal in tsetse flies: a risk for control campaigns? *Trends in Parasitology* **35**, 615-621. [**https://doi.org/10.1016/j.pt.2019.05.007**](https://doi.org/10.1016/j.pt.2019.05.007)
