# Supplementary material for: Negative density-dependent dispersal in tsetse (Glossina spp): An artefact of inappropriate analysis
Source: PLoS Negl Trop Dis. 2021 Mar 25;15(3):e0009026. doi: 10.1371/journal.pntd.0009026 (PMC8023489; doi:10.1371/journal.pntd.0009026)
Supplement: S3 Text — Data from Opiro (2017). Fig A in S3 Text. Distribution of estimates of N^e from the Opiro et al. (2017) study. Of the 42 study sites, only 30 provided finite estimates of N^e. (DOCX) [file pntd.0009026.s006.docx]

**Negative density-dependent dispersal in tsetse (Glossina spp):**

**an artefact of inappropriate analysis**

**J. W. Hargrove, J. Van Sickle, G. A. Vale, E. R. Lucas**

**S3 Text:**

**Procedure for “Thought Experiment” using data from Opiro (2017)**

**Multiplicative errors Calculation procedure**

With reference to Table B of S3 Table: “*Simulations used to estimate dispersal parameters where there may be errors of estimation for b, S and Ne*”, we provide the following description of our simulation procedure:

1. We carry out a “Thought Experiment” where we suppose that the Opiro et al. (2017) study, on *G. fuscipes fuscipes* in Northern Uganda is replicated 10 times, with different trap placements for each replicates.
2. At each of five of these sites, we assume that five traps are deployed on the circumference of a circle, and one at the centre of the circle, as depicted in Fig B(A) of S4 Text. The radius *r* of the circle, and thus *D_max_*, is varied between sites, and we assume that $\hat{S}$ = π *D_max_*^2^ can take values of 0.020, 0.08, 0.32, 0.64 or 2.56 km^2^, respectively.
3. In a further five sites, we assume that only one trap is used per site and sites are chosen such that the shortest distance (*D_min_*) between two sites can take values of 2, 6, 10, 14 or 20 km, respectively, giving values of $\hat{S}$ = π (*D_min_*/2)^2^ that range from 3 km^2^ to 314 km^2^. This type of trap deployment is depicted in Fig B(B) of S4 Text.
4. For all 10 replicates the sites are approximately uniformly distributed throughout the Opiro et al. (2017) study area.
5. With reference to Table B of S3 Table, the values of *D_max_* and *D_min_* are given in column B, and the resulting values of $\hat{S}$ in column C, with log($\hat{S}$) in column D.
6. For each of the 10 sampling procedures described above we suppose that the expected value of the effective population (*N_e_*) takes the value of 424.957 calculated by de Meeûs et al. (2019a). The values of *N_e_* and log(*N_e_*) are shown in columns E and F.
7. We allow that *N_e_* can be measured with, or without, error. Column G shows the stochastic error in log(*N_e_*), and the sum of columns F and G gives the estimated value of log($\hat{N}$*_e_*), in column H, and this is used to calculate $\hat{N}$*_e_* (column I).
8. The values of *D_e_* and $\hat{D}$*_e_* (columns J and L) are calculated from the appropriate values of *S* and *N_e_*, and $\hat{S}$ and $\hat{N}$*_e_*, and used to calculate log(*D_e_*) and log($\hat{D}$*_e_*), respectively (columns J to M).
9. In similar fashion, for each of the 10 sampling procedures described above we suppose that the expected value of *b* takes the value of 0.0202 used by de Meeûs et al. (2019a). The values of *b* and log(*b*) are shown in columns N and O.
10. We allow that *b* can also be measured with, or without, error. The error in log(*b*), and the resulting values of log($\hat{b}$) and $\hat{b}$ (columns P, Q, R) are generated as described above for the *N_e_*.
11. The values of *δ* (for situations where *N_e_* and *b* are measured without error) and $\hat{}$ (when there is stochastic error in $\hat{N}$*_e_* and $\hat{b}$) are shown in columns S and U, respectively – with their log values in columns T and V.
12. The original de Meeûs et al. (2019a) data (Table A of S3 Table – sheet 1) are used to produce the graphs in the top row of Fig A of S3 Table. The middle row shows the results for situation where *N_e_* and *b* are measured with stochastic error: and the bottom row results where *N_e_* and *b* are measured without error.
13. By hitting the F9 key, or otherwise refreshing the screen, the reader can make serial iterations of the realisations of the stochastic procedure – with each iteration using a different randomly generated errors for log($\hat{b}$) and for log($\hat{N}$*_e_*).

**Fig A.** **Distribution of estimates of** $\hat{\boldsymbol{N}}$**_e_ from the Opiro et al. (2017) study**.

Of the 42 study sites, only 30 provided finite estimates of $\hat{N}$_e_.
